# *Para*-Aminobenzoic Acid (PABA) Synthase Enhances Thermotolerance of Mushroom *Agaricus bisporus*

**DOI:** 10.1371/journal.pone.0091298

**Published:** 2014-03-10

**Authors:** Zhonglei Lu, Xiangxiang Kong, Zhaoming Lu, Meixiang Xiao, Meiyuan Chen, Liang Zhu, Yuemao Shen, Xiangyang Hu, Siyang Song

**Affiliations:** 1 State Key Laboratory of Stress Cell Biology, School of Life Science, Xiamen University, Xiamen, Fujian, China; 2 Key Laboratory of Biodiversity and Biogeography, Kunming Institute of Botany, Institute of Tibet Plateau Research at Kunming, Chinese Academy of Science, Kunming, Yunnan, China; 3 Edible Fungi Institute of Fujian Academy of Agricultural Sciences. Fuzhou, Fujian, China; 4 Department of Developmental and Molecular Biology, and Medicine, Albert Einstein College of Medicine, Bronx, NY, United States of America; Key Laboratory of Horticultural Plant Biology (MOE), China

## Abstract

Most mushrooms are thermo-sensitive to temperatures over 23°C, which greatly restricts their agricultural cultivation. Understanding mushroom’s innate heat-tolerance mechanisms may facilitate genetic improvements of their thermotolerance. *Agaricus bisporus* strain *02* is a relatively thermotolerant mushroom strain, while strain *8213* is quite thermo-sensitive. Here, we compared their responses at proteomic level to heat treatment at 33°C. We identified 73 proteins that are differentially expressed between *02* and *8213* or induced upon heat stress in strain *02* itself, 48 of which with a known identity. Among them, 4 proteins are constitutively more highly expressed in *02* than *8213*; and they can be further upregulated in response to heat stress in *02,* but not in *8213.* One protein is encoded by the *para*-aminobenzoic acid (PABA) synthase gene *Pabs*, which has been shown to scavenge the reactive oxygen species *in vitro*. *Pabs* mRNA and its chemical product PABA show similar heat stress induction pattern as PABA synthase protein and are more abundant in *02*, indicating transcriptional level upregulation of *Pabs* upon heat stress. A specific inhibitor of PABA synthesis impaired thermotolerance of *02*, while exogenous PABA or transgenic overexpression of *02* derived PABA synthase enhanced thermotolerance of *8213*. Furthermore, compared to *8213*, *02* accumulated less H_2_O_2_ but more defense-related proteins (e.g., HSPs and Chitinase) under heat stress. Together, these results demonstrate a role of PABA in enhancing mushroom thermotolerance by removing H_2_O_2_ and elevating defense-related proteins.

## Introduction

*Agaricus bisporus*, also called button mushroom, is one of the most popular edible basidiomycete fungus worldwide and well-known for its low content of fat and high content of dietary cellulose and pharmacological components [1], [2]. So far, *A. bisporus* has been agriculturally cultivated in more than 100 countries, especially in China, and has already become one of the most prevailing species of dietary mushroom [3]. Like other mushrooms, *A. bisporus* is thermo-sensitive and vulnerable to high temperatures. Once the ambient temperature exceeds 23°C, *A. bisporus* fruit body grows slowly and its sporophores become smaller and brown, which greatly affects the quality and yield in cultivation. In order to ensure a large scale off-season cultivation of this commercially important species, special cooling instruments and more energy expense are required to maintain the ambient temperature below 20°C, which has become the major restrictive factor in mushroom industry worldwide [4]. Therefore, understanding the intrinsic mechanisms of acquired thermotolerance of mushrooms under heat stress is of both theoretical and practical significance, and eventually contributes to the mushroom cultivation industry.

Organisms use different ways to respond to environmental stress such as high temperature. One of the best characterized factors is the heat shock protein (HSP) family, which plays a central role in thermotolerance of plants and animals [5]. Three major classes of HSPs are present in organisms including the small HSPs (ranging in molecular weight from 15 to 28 kD), the moderate HSPs (such as HSP60 and HSP70) and high HSPs (such as HSP90 and HSP101). Most HSPs can ameliorate protein misfolding and aggregation during heat stress, but each major HSP family uses a distinct and rigorous mechanism to prevent heat stress induced impairment. For example, Hsp60 and HSP70 can prevent protein aggregation by binding to protein intermediates, while HSP101 can reactivate a protein that has already aggregated. In addition to HSPs, many other factors, such as plant hormone ABA, reactive oxygen and salicylic acid, are also involved in acquired thermotolerance [6], [7], [8], [9]. However, very little is known about the thermotolerant signaling in higher basidiomycetes.

PABA (*para*-aminobenzoic acid), also known as 4-aminobenzoic acid, is a precursor for the synthesis of folic acid (also known as vitamin B_9_ or folacin). As an enzyme cofactor, folic acid is involved in numerous basic biological reactions, including nucleotide biosynthesis, DNA repair and DNA methylation. For example, children and adults both require abundant folic acid to produce healthy erythrocytes and avoid anemia [10]. As the primary source of folic acid [11], plants firstly synthesize the intermediate PABA in chloroplasts via PABA synthase and then use PABA for folic acid synthesis in mitochondria [12], [13], [14]. Most previous studies focused on the role of the PABA metabolite in folic acid synthesis. Interestingly, salicylic acid (SA), an analog of PABA, is well known for its wide range of functions from human pain relief to plant system defenses [15]. In recent years, SA has been found to protect Arabidopsis, tomato, bean [16], potato, mustard and tobacco against heat stress [9], [17]. Due to the structural similarities between PABA and SA, it is highly possible that PABA may also play a role mediating plant thermotolerance response, but its roles in plants responding to environmental stress remain elusive.

In this study, we set out to identify novel signaling pathways and novel secondary messengers that are involved in thermotolerance of mushroom against heat stress. We started by performing comparative proteomic profiling on two *A. bisporus* strains, *8213* and *02*, which are phylogenetically closely related, but otherwise completely different in thermotolerance. For mycelium growth, strain *8213* (will be called *8213* herein) is thermo-sensitive and vulnerable to high temperatures over 33°C, while strain *02* (will be called *02* herein) is thermotolerant to such temperature. By employing 2D-electrophoresis, a set of proteins differentially expressed between *8213* and *02*, with or without heat stress, were identified. Among them, one protein encoding mushroom PABA synthase was constitutively more abundant in *02* than in *8213* at both mRNA and protein level, and was further upregulated under heat stress in *02*. Further physiological and transgenic experiments demonstrated that PABA may be a messenger that mediates mushroom thermotolerance response. Our findings provide insights into the role of PABA in mushrooms thermotolerance and implicate a novel target for transgenic improvement of mushroom thermotolerance.

## Materials and Methods

### Mushroom Strains and their Growth Conditions

Mushroom (*A. bisporus*) strains *02* and *8213* were provided by the Mushroom Research and Development Station, Fujian Academy of Agricultural Sciences, China. They were cultured at 23°C on a potato dextrose agar (PDA) plate for two weeks to obtain mycelium growth. The fruit body cultivation was performed at 18°C as previously described [18].

### Thermotolerance Assay

The heat sensitivity of mushroom strains was determined primarily by the mycelium elongation assay. The well-grown mycelia were inoculated into a test tube that contained a cottonseed hull medium (78% cottonseed hull, 15% cow dung, 5% wheat bran, 2% plaster and lime mixture). The test tubes were kept vertical throughout this test. After 5 days of normal growth at temperatures at 23°C, the mycelium elongation position of each test-tube was marked as the starting point. Subsequently, the experimental groups were subjected to 33°C heat stress conditions. Mycelium elongation of each test tube was marked every 7 days, and the elongation length was recorded. In this assay, we used the mycelium elongation length to quantify heat sensitivity of strains *02, 8213* and each transgenic line. Furthermore, we used the hypha damage observation and the fruit body formation as supplements to thermotolerance determination that provided similar conclusions [19], [20].

### Thermotolerance Induction and Sample Collecting

After growing in 100 ml liquid PDA medium in 250-ml Erlenmeyer flasks for 2 weeks at 23°C on a rotary shaker (160 rpm), the mycelia culture of strains *02* and *8213* were divided into heat treatment and control groups. For the heat treatment groups, both strains were exposed to the stress temperature of 33°C for 24 h. Controls strains were cultivated for the same period of time at 23°C. After heat treatments, the mycelia were collected, frozen in liquid nitrogen immediately and stored at −70°C. The analysis of proteomes was carried out for the two comparative groups.

### Two-DE Proteome Profiling and Image Analysis

Mushroom mycelium samples collected from each experimental group were ground to a powder in liquid nitrogen. Total protein extraction and quantification were conducted according to previously described methods [21]. In brief, the samples were extracted with a dehydration buffer (2 M thiourea, 7 M urea, 2% CHAPS, 40 mM DTT, 0.002% bromophenol blue, 2% IPG buffer), and stored at −70°C. Each protein sample (500 µg) was loaded on an IPG (immobilized pH gradient) gel for 12 h at 20°C for in-gel rehydration. The first-dimension IEF was carried out with 18 cm-long, pH 3–10, non-linear Immobiline DryStrips (GE Biosciences) and performed on an IPGphor (Amersham) system using the following program: 500 V for 1 h, 1000 V for 1 h, and 8000 V for 8 h 20 min (total 68,000 Vh). The focused IPG strips were placed in equilibrium buffer (75 mM of Tris-HCl, pH 8.8, 6 M urea, 29.3% v/v glycerol, 2% w/v SDS, and 0.002% bromophenol blue) containing 10 mg/ml of DTT for 15 min followed by another 15 min treatment in the same buffer containing 25 mg/ml iodoacetamide. The second dimension electrophoresis was carried out using a 12% SDS-PAGE to separate proteins by molecular weight.

All gels were stained with colloidal Coomassie Brilliant blue G-250 and scanned by an image scanner with a resolution of 300 dpi. Image analysis was carried out with Image-Master 2D Platinum version 5 software (GE Biosciences). Each spot was assigned a value as the volume of differential expression (V_DE_), which is defined as the sum of the gray-level values of all the pixels in the spot. Three biological replicates were used to calculate the average values. Candidate spots exhibiting 2-fold changes or greater were selected for identification, and the Student’s *t*-test was performed to calculate statistical significance.

### In-gel Digestion

Protein spots were excised from the gel above and washed 3 times with ultrapure water, and then destained twice with 50% acetone in 25 mM NH_4_HCO_3_. The gels were dehydrated with 50 µL 100% acetonitrile, lyophilized and further digested overnight with trypsin (Promega, Madison, WI, US) in 25 mM NH4HCO3 containing 10% CAN at 37°C. For each spot, the supernatant was transferred to a separate Eppendorf tube and lyophilized for MS analysis.

### MALDI-TOF (Matrix-Assisted Laser Desorption/Ionization Time-of-Flight)/TOF Analysis and Database Search

Tryptic-digested peptides were extracted according to a previous protocol [21], and MS analysis was performed with a MALDI-TOF/TOF mass spectrometer (Bruker-Franzen Analytik) in reflectron mode with an accelerating voltage of 25 kV and a delayed extraction of 50 ns. Mass spectra were obtained in an automatic mode with the AutoXecute module of Flexcontrol (Bruker-Franzen Analytik) (laser power ranged from 40 to 60%, 1000 shots). Flex-Analysis software (Bruker-Franzen Analytik) was employed to analyze the spectra with the auto proteolysis peptides of trypsin (*m*/*z* 842.51, 1045.56, 2211.10) as the internal calibration. Peptides were selected in the mass range of 800–3500 Da. For each sample, 4–5 ion peaks with signal-to-noise ratios over 100 were selected as precursors for secondary mass spectrum analysis, and the TOF/TOF signal for each precursor was accumulated with 2000 laser points. The peptide MS/MS spectra were compared against the domestic database download from the public mushroom web site (http://genome.jgi-psf.org/Agabi_varbisH97_2/Agabi_varbisH97_2.home.html) using MASCOT software (http://www.matrixscience.com). Fixed modification was set for the carbamidomethylation of cysteine, while the oxidation of methionine was set as a viable modification. The peptide tolerance was 60 ppm, and the MS/MS tolerance was 0.25 Da. One incomplete cleavage was allowed for each protein. Keratin contamination was removed, and a MASCOT score over 60 (p<0.05) was considered to be a positive hit.

### Transgenic Manipulation

For mushroom transgenic manipulation, the ORF of *A. bisporus* PABA synthase gene (*Pabs*) was PCR-amplified from the cDNA of strain *02* and subsequently cloned into the binary vector pBHg provided by the Mushroom Research and Development Station, Fujian Academy of Agricultural Sciences, China. The construct was then transformed into strain *8213* using the Agrobacterium mediated gill-soaking method as described previously [18]. At least 10 independent lines were selectively grown on a PDA plate containing 50 mg l^−1^ hygromycin for further analysis.

### Quantitative Real-time PCR Analysis

Total RNA was extracted, DNaseI digested and reverse transcribed for use as a template. SYBR Green dye and gene-specific primers were added to perform the real-time PCR quantification with a LightCycler real-time PCR system (ROCHE). A melting curve analysis of each gene was performed to check the specificity of amplification, and Gapdh was used as a reference control and corrected for PCR efficiency differences between target and reference samples with efficiency correction Relative Quantification Software (LightCycler Software 4.05). Each of the amplifications was performed in duplicate, and the mean value was calculated as the final result.

### Total PABA Extraction and HPLC Quantification

Total PABA extraction was performed as described by Eoin P. Quinlivan [22] with some modification. Briefly, mycelium (1 g of fresh weight) was ground in liquid nitrogen and extracted twice with 3 ml of methanol. The two parts of the supernatant were then pooled and evaporated. The dried sample was dissolved in 1 ml of 0.1 M sodium acetate buffer, pH 5.5, and digested with 0.04 U/µl glycosidase (Sigma) at 30°C overnight to change the conjugated PABA into free type. Subsequently, the product was evaporated, re-dissolved in 0.8 ml of 0.1 M sodium citrate, pH 3.7, and partitioned against 2.4 ml of ethyl acetate (EA). PABA was recovered from the organic phase by back extraction with 0.7 ml of 50 mM NaOH and neutralized. After final evaporation, the sample was dissolved in methanol solvent.

The extracted sample was then injected onto a 5 µm C18 (250 × 4.6 mm, Agilent) column and detected by fluorescence (270 nm excitation, 350 nm emission). The column was eluted (1 ml/min) with 0.5% acetic acid/methanol (80∶20, v/v) [23]. The total PABA (free PABA and conjugated PABA) was quantified according to the standards. The spiked PABA sample was also quantified for the recovery rate correction.

### H_2_O_2_ Concentration and Antioxidant Enzymes Activities Measurement

The mushroom mycelium (0.5 g) were quickly frozen in liquid Nitrogen and ground to a fine powder. The frozen powdered samples were then homogenized in 5 mL of extraction buffer (10 mM Tris-Cl, pH 8.0, 1 mM phenylmethylsulfonyl fluoride, 10 mM MgSO_4_, 5 mM KCl, 5 mM NaCl, 10 µM oxyhemoglobin and 10 units ml^−1^ of catalase). The extracts were then centrifuged (15,000×*g*, 10 min) at 4°C, and the supernatants were used for H_2_O_2_ determination; H_2_O_2_ content was measured using a previously described method [24]. The antioxidant enzymes including Catalase and SOD enzyme activities were measured as previous method [24].

### Western Blotting Analysis

With liquid nitrogen grounding, total protein from mycelium was extracted and dissolved in SDS sample buffer as described. Then the protein concentration of each sample was determined using Coomassie Blue assay, and diluted to equal. After 15% (w/v) SDS-PAGE separation, the proteins were blotted onto PVDF membrane and immunoblotted against the anti-HSP27 (cell signaling) and anti-HSP70 (Stressgen) respectively with a dilution of 1∶1,000 (v/v). The antibodies against mushroom PABA synthase and Chitinase were prepared by immunizing the rabbit with the purified PABA synthase protein or synthesized peptides from mushroom Chitinase (VTFNGHLWQNK).

## Results

### *Agaricus bisporus* Strain *02* is more Thermotolerant than Strain *8213*

**Figure 1:**
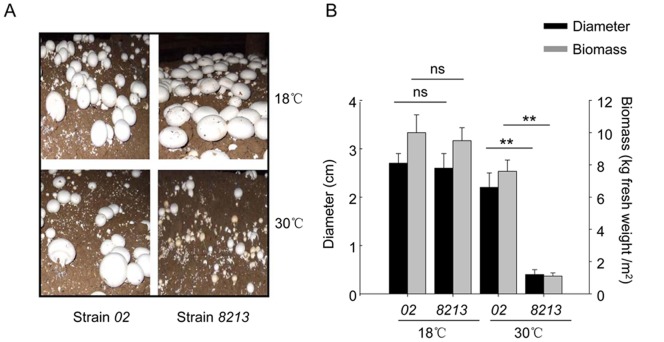
Mushroom strain *02* is more thermotolerant than strain *8213*. Strains *02* and *8213* had been cultivated under the same regular condition until about 1 week before fruit body formation, each was then divided into two groups. One half was maintained at 18°C (control group); the other half was maintained at 30°C (high-temperature group) for 7 days. At Day 7, the fruit bodies were photographed (A), and the pileus diameter and biomass weight were recorded (B). Three independent replicates were performed. Data are expressed as average ± SEM. Unpaired t-tests were performed, ns: P>0.05, **: P<0.01.

*Agaricus bisporus* strain *02* is one of the common mushroom strains that are used in agriculture and research. Strain *02* is characterized as a thermotolerant strain. Mycelium growth of *02* is resistant to an ambient temperature over 33°C, which is a lethal temperature for most of the other common strains. In contrast, during our breeding practice, we screened out strain *8213* that was significantly more sensitive to heat stress than regular *A. bisporus* strains. As shown in Figure 1A & 1B, at 18°C, a regular ambient temperature for mushroom cultivation, the fruit bodies of *02* and *8213* grew similarly well. When the ambient temperature rose to 30°C, the fruit body growth of *8213* was severely reduced in both pileus diameter and biomass, while the *02* still had a comparable growth of biomass as at 18°C. Phylogenetic analysis showed that *8213* and *02* both belong to the same species *Agaricus bisporus*, and *02* is evolutionally closer to *8213* than other strains of *A. bisporus* (Figure S1). Thus, *02* and *8213* provide a pair of experimental models for studying the mechanisms of intrinsic thermotolerance in mushroom.

### Proteomic Profiling of *02* and *8213* Reveals a Potential Role of PABA-synthase in Enhancing Thermotolerance

To investigate the mechanisms underlying higher thermotolerance of *02*, we employed comparative 2-dimensional electrophoresis (2-DE) to profile the proteomics difference between *02* and *8213* with or without heat stress.

To avoid inducing drought stress, we choose PDA liquid culture for mushroom mycelia growth. We first determined optimal heat stress condition for our studies. The optimum temperature for mushroom mycelium growth in liquid culture is 23°C; we tested the thermo-sensitive strain *8213* in a set of temperatures (23°C, 33°C, and 43°C) for 48 h, and then inoculated them separately into fresh PDA culture for another week of growth at 23°C. We found that mycelia treated with 43°C failed to re-grow in fresh medium, while mycelia treated with 33°C grew to reduced biomass of about 70% of the 23°C treated group, suggesting that 33°C may be a appropriate temperature for heat stress. We then treated strain *02* with 33°C for a time course (12 h, 24 h, and 48 h) and used the heat shock proteins (HSP20 and HSP70) as markers to determine the best time point for proteomic profiling. As a result, we found that both Hsp20 and Hsp70 began to show accumulation at 48 h time point, without any noticeable accumulation at 24 h time point, which suggested that, under 33°C heat stress condition, the upregulation of those heat stress terminal effectors happens in the time gap between 24 h to 48 h (data not shown). In order to better profile proteins that govern the expression of HSPs, we used 33°C for 24 h as the heat stress condition for proteomic profiling.

**Figure 2:**
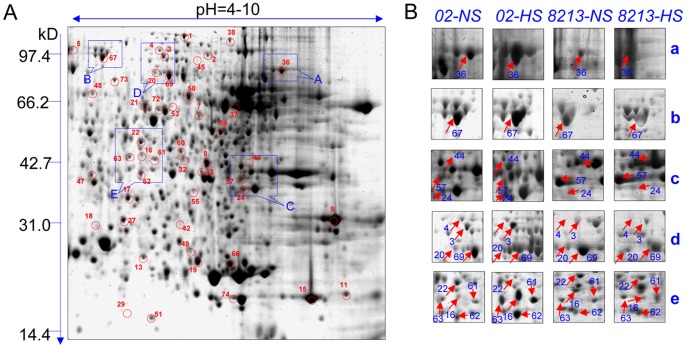
2-D electrophoresis of protein extracts from *02* and *8213* with or without heat stress (33°C/24 h). (A) Representative 2-DE gels of mushrooms, identifying 73 proteins with a greater than 2-fold difference after high-temperature treatment (p<0.05). Molecular weight (MW) in kilodaltons and pI of proteins are indicated on the left and top of the gel, respectively. (B) Close-up view of some differentially expressed proteins spots. Three independent replicates were performed. *02*-NS: *02* non-stressed (23°C/24 h); *02*-HS: *02* heat-stressed (33°C/24 h); *8213*-NS: *8213* non-stressed (23°C/24 h); *8213*-HS: strain *8213* heat-stressed (33°C/24 h).

Strains *02* and *8213* were treated with or without the heat stress condition determined above. Proteins were isolated from their mycelia and resolved by 2-DE. For brevity, we will use *02*-NS and *02*-HS to indicate non-stressed strain *02* and heat-stressed *02*, respectively; and use *8213*-NS and *8213*-HS to indicate non-stressed strain *8213* and heat-stressed *8213*, respectively. Figure 2 and Figure S2 show the results for strain *02* and *8213* under normal condition (23°C) and heat stress condition (33°C), respectively. The gels were stained by CBB R-50, scanned and analyzed with Image-Master 2D Platinum version.5 software (GE Biosciences). We found a total of 340 protein spots showing differential expression in one of the three pairs of comparisons (*02*-NS vs. *02*-HS, *8213*-NS vs. *02*-NS and *8213*-HS vs. *02*-HS). Among them, 73 spots reach the criterion of 2-fold change with statistically significance (P<0.05). Those 73 spots were then subjected to MS/MS analysis and peptide mass fingerprinting (PMF) identification in the NCBI database. The identities of 48 proteins were successfully determined (Table S1).

**Figure 3:**
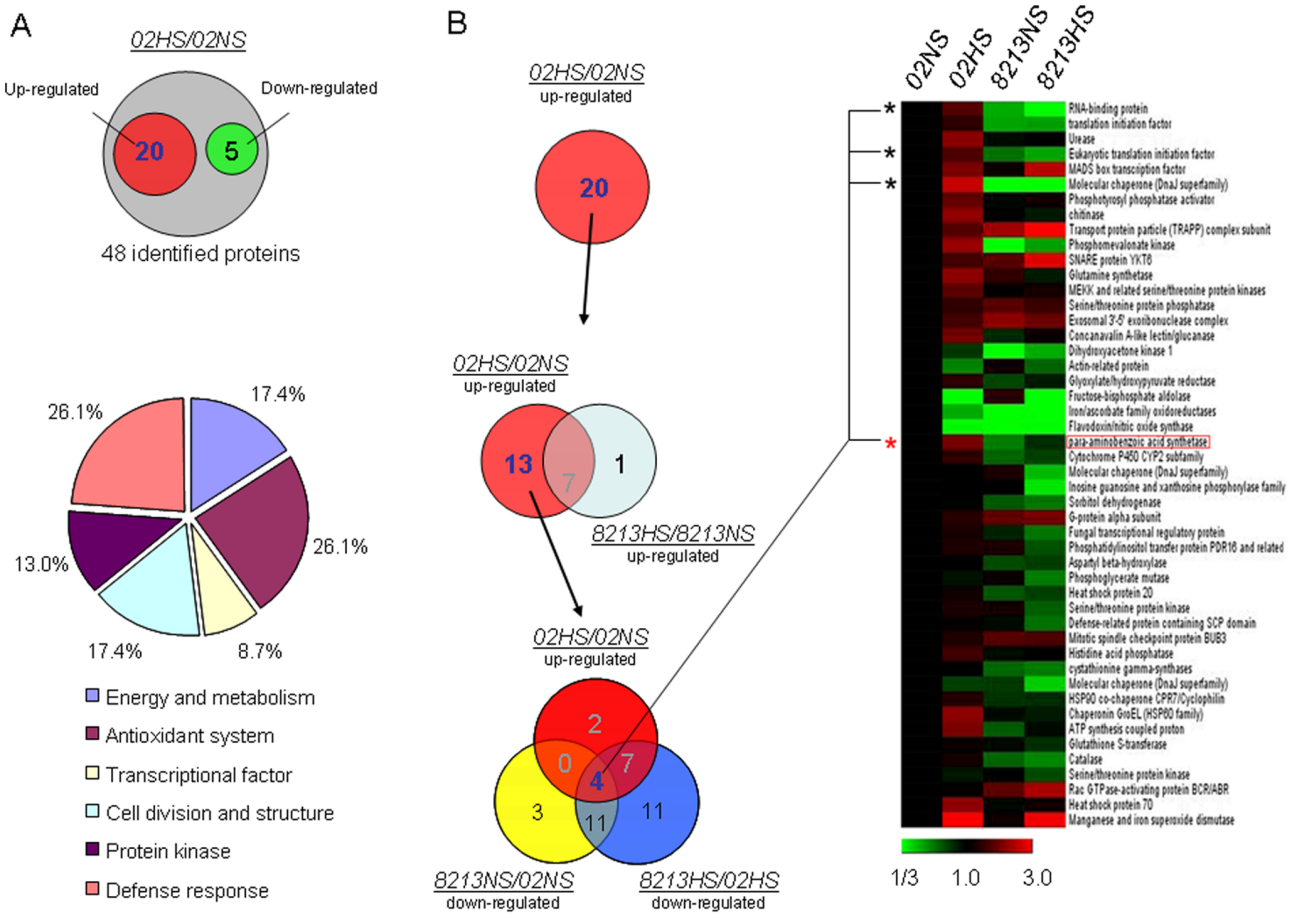
Identification and classification of differentially expressed proteins between *02* and *8213*. (A) Venn diagram showing 20 up-regulated and 5 down-regulated proteins among 48 identified proteins comparing *02*-HS with *02*-NS. Functional classification of those 25 heat stress induced proteins is shown on the bottom. (B) Heat-map is shown to indicate relative protein abundance among the 4 samples. The abundance of each protein spot in *02*-NS was given a reference value of 1.0, the abundance of that spot from *02*-HS, *8213*-NS and *8213*-HS were transformed into relative value. The different colors correspond to the values of protein level changes as indicated by the bar at the bottom of the heat map. Venn diagrams present the two-step filtering process leading to 4 candidates to further select out the more important candidate proteins for functional studies (see text). The four asterisks mark the proteins that are upregulated by heat-stress in thermotolerant strain *02*, not upregulated by heat stress in thermo-sensitive strain *8213,* and expressed at lower levels in thermo-sensitive strain *8213* than in the thermotolerant strain *02* with or without heat-stress. *02*-NS: *02* non-stressed (23°C/24 h); *02*-HS: *02* heat-stressed (33°C/24 h); *8213*-NS: *8213* non-stressed (23°C/24 h); *8213*-HS: strain *8213* heat-stressed (33°C/24 h).

We set the abundance of each protein spot in *02*-NS as the reference value of 1.0, and then converted the protein abundance of that spot from *02*-HS, *8213*-NS and *8213*-HS gels into relative value. We generated heatmap for all the 48 proteins between all 4 samples and found that 25 out of 48 spots shows changed expression level (>2.0 fold) in thermotolerant strain *02* upon heat stress (Figure 3A top). Based on their predicted metabolic and functional features (using the COGNITOR tool in Cluster of Orthologous Groups (COG) database), those 25 proteins could be classified into six groups as shown in Figure.3A. Among the 25 proteins, 20 spots show up-regulation, while the other 5 spots show down-regulation, indicating that upregulated proteins are more likely to be responsible for thermotolerance enhancement in *02*. We decided to focus on those 20 up-regulated proteins for further studies. Most of the up-regulated proteins in *02* upon heat stress belong to the defense response group (26.1%) or the antioxidant protein family (26.1%) (Figure 3A bottom). Since strain *02* is a thermotolerant strain and is also the center of this study, we next screened out candidate genes that are responsive to heat-stress in *02* but not in *8213*, and are expressed higher in *02* than in *8213* in non-stressed conditions. Severn out of 20 spots were also upregulated (>2.0 fold) in *8213* after heat stress, and were excluded (since their up-regulation did not confer thermotolerance to *8213*) (Figure 3B, middle). For the remaining 13 proteins, we excluded those that are not expressed higher in strain *02* over *8213* either in non-stress condition or in heat stress conditions, leaving 4 proteins as our candidates (Figure 3B, bottom).

**Figure 4:**
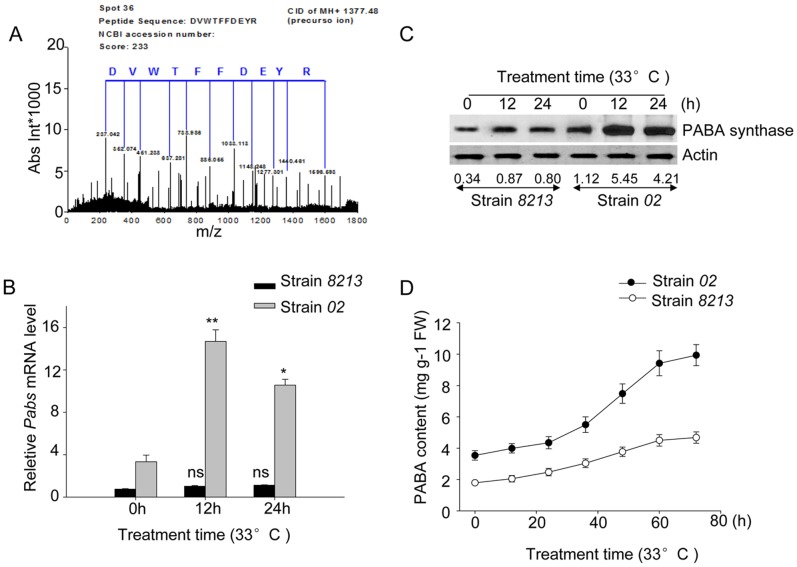
Thermotolerant strain *02* responded to heat stress with more PABA synthesis than strain *8213*. (A) MS/MS analysis identifies the protein spot 36 to be mushroom PABA synthase. (B) Differential transcription of PABA synthase gene *Pabs* in strain *02* and strain *8213* with or without heat stress. Strain *02* and strain *8213* were treated with high temperature at 33°C for the indicated time and the mRNA level of *Pabs* was quantified by real-time PCR. Three independent biological replicates were performed. Data are expressed as average ± SEM. Unpaired t-tests were performed between high temperature treatment samples and control samples (without treatment) within each strain, respectively, ns: P>0.05, *: P<0.05, **: P<0.01. (C) Differential protein accumulation of PABA synthase in strain *02* and strain *8213* after heat stress. Strain *02* and strain *8213* were treated with high temperature at 33°C for the indicated time, and PABA synthase protein levels were measured by western blot as indicated. (D) Differential accumulation of PABA content in strain *02* and strain *8213* after heat stress. Strain *02* and strain *8213* were treated with high temperature at 33°C for the indicated time and the content of PABA was measured by HPLC. Three independent biological replicates were performed. Data are expressed as average ± SEM.

Among these four proteins, protein spot 36 (Figure 2B-a) was indentified by MS/MS spectra peak analysis to encode a mushroom *para*-aminobenzoic acid (PABA) synthase (Figure 4A). Consistently, the EST fragment of PABA synthase gene (*Pabs*) was found to be more highly expressed in strain *02* than in *8213* in our suppressive subtractive hybridization (SSH) (EST Genbank accession#: GH159019.1). Collectively, these studies identify PABA synthase as likely playing a role in enhancing thermotolerance of strain *02*.

### PABA Synthesis is Stimulated in Thermotolerant Strain *02* under Heat Stress

By performing the RACE technique, we cloned the *Pabs* gene ORF sequence in mushroom (GenBank accession#: FJ617437), and determined that its full-length open reading frame (ORF) is 1800 bp and encodes a 600-amino acid PABA synthase.

To determine whether the accumulation of PABA synthase is due to transcriptional level induction upon heat stress or due to changes in protein stability, we perform qRT-PCR experiment with mRNA samples of the corresponding sets of mushroom mycelia. Consistent with the pattern of protein level change, the PABA synthase gene (*Pabs*) constitutively expresses higher in *02* than in *8213* and upregulates dramatically in strain *02*, but only slightly changes in strain *8213,* under heat stress (Figure 4B). Thus, transcriptional regulation of the *Pabs* gene likely accounts for its protein level changes in *02* and *8213* in non-stress and heat stress conditions.

To study the functions of *Pabs* in thermotolerance, we sub-cloned its ORF into the pGEX-4T1, expressed the protein in *E.coli BL-21* strain (Figure S3), and purified the protein for enzymatic analysis. Our results showed that purified PABA synthase protein exhibited a considerable PABA synthesizing activity *in vitro* (data not shown). We then generated antibody against PABA-synthase by immunizing the rabbit with the bacterially expressed PABA synthase protein. With this antibody, we confirmed that the endogenous PABA synthase significantly increased over time under heat stress in *02*, but not in *8213* (Figure 4C).

Since PABA synthase is expected to synthesize PABA in mushroom, we also measured the PABA content in *02* and *8213* with or without high-temperature treatment. Results showed that PABA level was less than 2 fold higher in *02* than in *8213* at the onset of high-temperature treatment. After 72 h of high-temperature treatment, PABA content increased dramatically in *02*, reaching to around 10 mg/g FW, while to around 3 mg/g FW in *8213* treated in parallel (Figure 4D).

### Increasing PABA Enhances Thermotolerance of the Thermo-sensitive Strain *8213*

**Figure 5:**
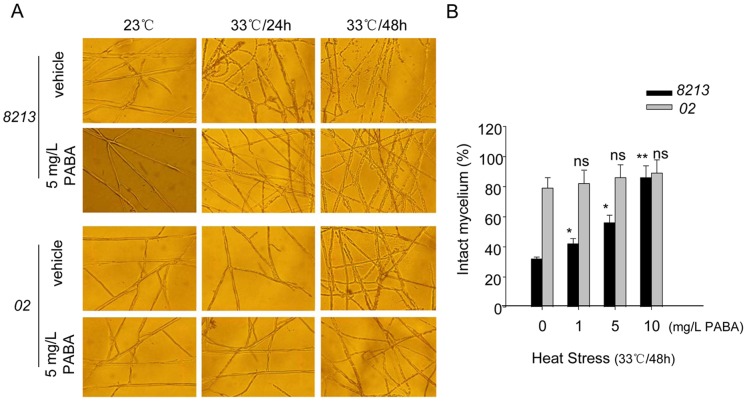
Exogenous PABA improves thermotolerance of strain *8213*. (A) Strains *02* and *8213* mycelium culture were supplied with or without 5 mg/L PABA, followed by high temperature treatment at 33°C for 48 hours, the images were taken at the indicated time post-heat stress. The control groups were cultured under regular temperature of 23°C. (B) Dose-dependent protection by PABA of mycelium intactness under heat stress at 33°C for 48 hours. Strain *02* and strain *8213* were treated with different concentrations of PABA followed by high-temperature treatment at 33°C for 48 h, and the percentage of intact mycelia was counted. The intact mycelia percentage was calculated as compared to the control group (23°C). Three independent biological replicates were performed. Data are expressed as average ± SEM. Unpaired t-tests were performed between samples with and without PABA addition within each strain, respectively, ns: P>0.05, *: P<0.05, **: P<0.01.

To determine the role of PABA in mushrooms undergoing high-temperature stress, we compared the responses of *02* and *8213* to exogenous addition of PABA to the culture medium. We used the mycelium breakage as the criteria for sensitivity of mushrooms to high temperatures because mushroom mycelium easily breaks and cannot grow well under high temperatures. As shown in Figure 5A, for strain *8213*, most mycelia broke under 33°C after 48 hours, as compared to control sets which were cultured under regular 23°C. Addition of PABA to medium alleviated the mycelium breakage caused by high temperatures, and this alleviation effect was even more obvious at 10 mg/L PABA concentration in the medium. For strain *02*, addition of exogenous PABA did not further improve its thermotolerance (Figure 5A&5B), suggesting that the endogenous levels of PABA in *02* might approach saturating point.

**Figure 6:**
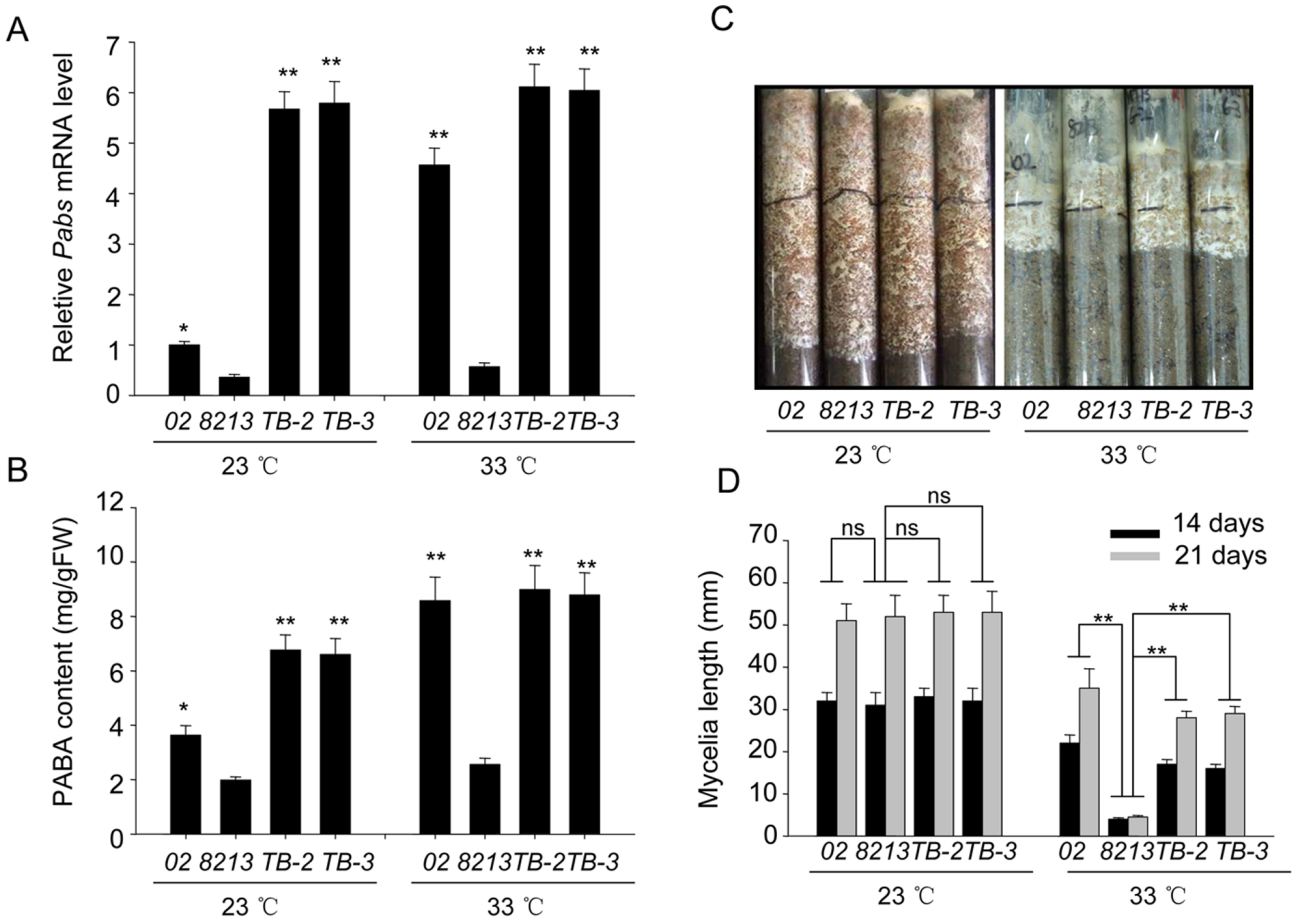
Transgenic overexpression of PABA synthase improves thermotolerance of strain *8213*. (A) Relative mRNA level of *Pabs* gene of strains *02*, *8213* and two *Pabs*-overexpressing transgenic strains *TB-2* and *TB-3* (derived from *8213*) under normal temperature (23°C) and heat stress (33°C). The mRNA of corresponding samples was extracted and analyzed after 24 hours of treatment. (B) The PABA content of strains *02*, *8213* and *TB-2* and *TB-3* under normal temperature (23°C) and heat stress (33°C) for 3 days. The PABA content of corresponding samples was extracted and measured after 3 days of treatment. (C) The mycelia growth of Strains *02*, *8213* and *TB-2* and *TB-3* under normal temperature (23°C) and heat stress (33°C). Mycelia cultures were photographed after 2 weeks of treatment. (D) The mycelia elongation of strains *02*, *8213* and *TB-2* and *TB-3* under normal temperature (23°C) and heat stress (33°C). The mycelia length is measured after 14 and 21 days of treatment. Three independent biological replicates were performed for each analysis. Data are expressed as average ± SEM. Unpaired t-tests were performed between strain *8213* and all other strains as indicated within each treatment condition, ns: P>0.05, *: P<0.05, **: P<0.01.

Since thermotolerant strain *02* has higher level of endogenous PABA synthase, and addition of PABA in medium helped to enhance thermotolerance of the thermo-sensitive strain *8213*, we further tested whether transgenic over-expression of PABA synthase would confer *8213* with higher thermotolerance. We subcloned the *02*-derived *Pabs* ORF into the intermediate vector pBHg, which uses a constitutive mushroom *Gapdh* promoter to drive the transcription of target gene *in vivo*. Then we introduced the *Pabs* expressing plasmid into thermo-sensitive strain *8213* using agobacteria mediated transformation, and obtained several independent transgenic lines by antibiotic selection. By using real time qPCR to examine the transcriptional level of *Pabs* in these transgenic strains, we obtained and confirmed two transgenic lines, named *TB-2* and *TB-3*, that constitutively express higher levels of *Pabs* (23°C), which is 5 fold higher than the constitutive *Pabs* level in strain *02* (Figure 6A). As shown, although the transcription of *Pabs* in *TB-2* and *TB-3* do not upregulate upon heat stress, their abundance is still higher than the induced *Pabs* level of strain *02* under heat stress (33°C). We further measured the endogenous PABA content of *02* and *8213* as well as the two transgenic strains. Indeed, *TB-2* and *TB-3* contained higher level of PABA in both normal (23°C) and heat stress (33°C) conditions compared to strain *8213* (Figure 6B). After validating the effectiveness of the transgenic manipulation, we determined the effects of overexpression of *Pabs* on thermotolerance. In Figure 6C and 6D, we used the mycelia elongation essay to quantitatively compare thermotolerance capability between those mushroom strains. All strains grew well under normal conditions (23°C), as measured by their similar mycelia elongation length. Under heat stress condition, the mycelia growth of *8213* was almost completely abolished, while *02* continued to grow although at a slightly reduced rate. Interestingly, *TB-2* and *TB-3*, which originated from *8213* but constitutively overexpress *02*-derived *Pabs* gene, showed similar mycelia growth as *02* (Figure 6D).

Collectively, these studies demonstrate that gain-of-function of *02*-derived *Pabs* gene in thermo-sensitive strain *8213* enhanced its thermotolerance capability.

### Suppressing PABA Synthesis Compromises Thermotolerance of the Thermotolerant strain *02*

**Figure 7:**
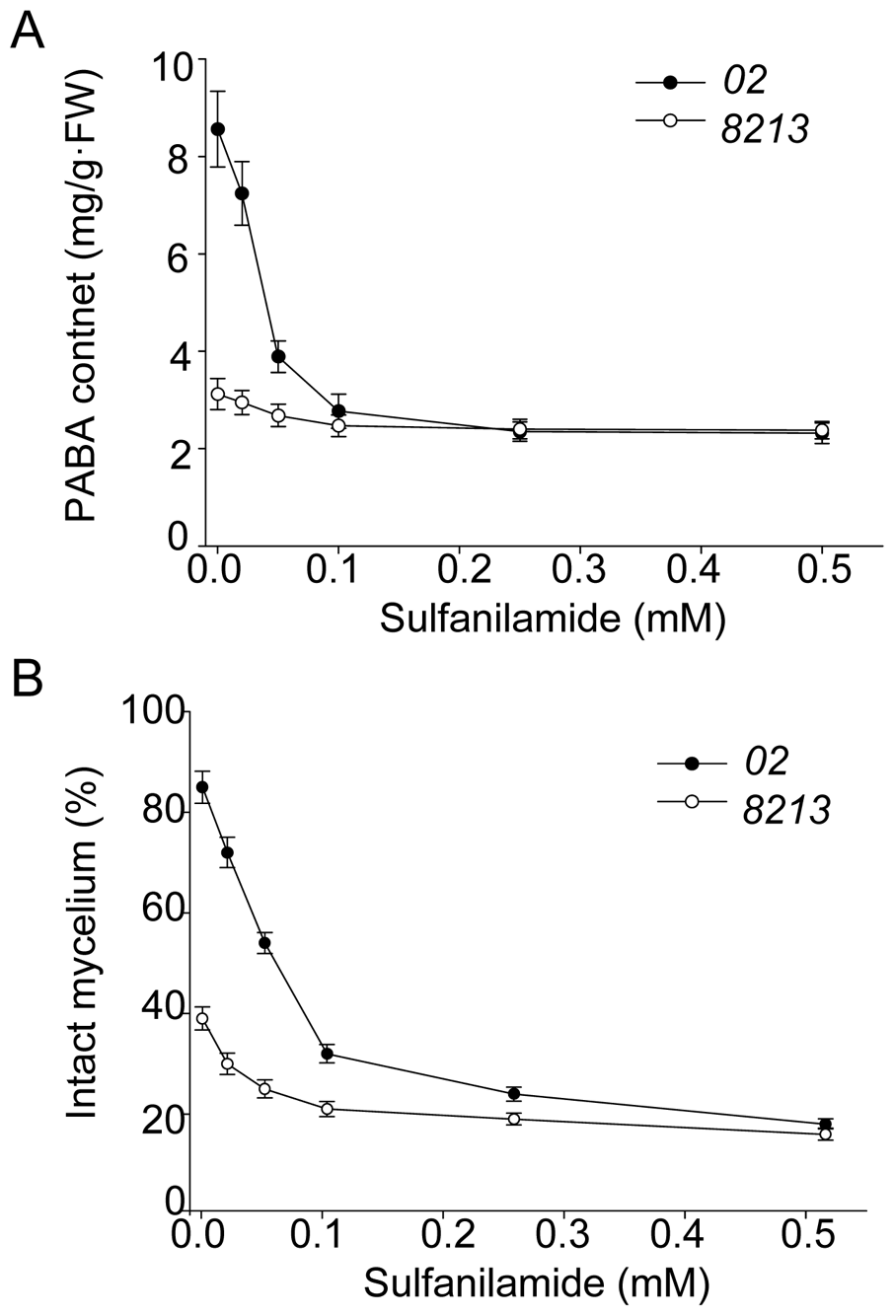
PABA synthase inhibitor sulfanilamide inhibits PABA production and impairs mycelium intactness. Mushrooms were treated with increasing concentrations of sulfanilamide for 6 days, and then subjected to heat stress (33°C) for 48 hours. PABA content (A) and the percentage of intact mycelium (B) were determined after heat stress treatment.

PABA synthesis and utilization can be suppressed by sulfanilamide, a structural analog of PABA [25]; and strain *02* has higher basal and induced level of *Pabs* gene and PABA production. To verify the relationship between PABA function and thermotolerance, we treated *02* with sulfanilamide to carry out loss-of-function studies. We found that addition of sulfanilamide reduced PABA production in *02* under high temperature (Figure 7A). Without the inhibitor, strain *02* contained PABA content around 9 mg/g FW under heat stress; 0.1 mM sulfanilamide treatment abolished about 70% of its PABA production. This effect reached saturation at concentrations over 0.25 mM. After validating the inhibitory effect of sulfanilamide on PABA production, we investigated whether reduction of PABA content in strain *02* would compromise its thermotolerance. Indeed, the percentage of intact mycelia of *02* dramatically decreased following treatment of 0.25 mM sulfanilamide for 48 hours (Figure 7B). The biomass of *02* was also reduced by sulfanilamide treatment (data not shown). The same sulfanilamide treatment did not further aggravate the damage seen in strain *8213*, likely because *8213* natively express less PABA and has less intact mycelium under heat stress. Thus, inhibition of PABA synthase dramatically compromised thermotolerance capability of thermotolerant strain *02*, providing further evidence for the relationship between PABA synthase function and thermotolerance.

### PABA Alleviates Oxidative Damage by Heat Stress through Increasing the Antioxidases Activity

**Figure 8:**
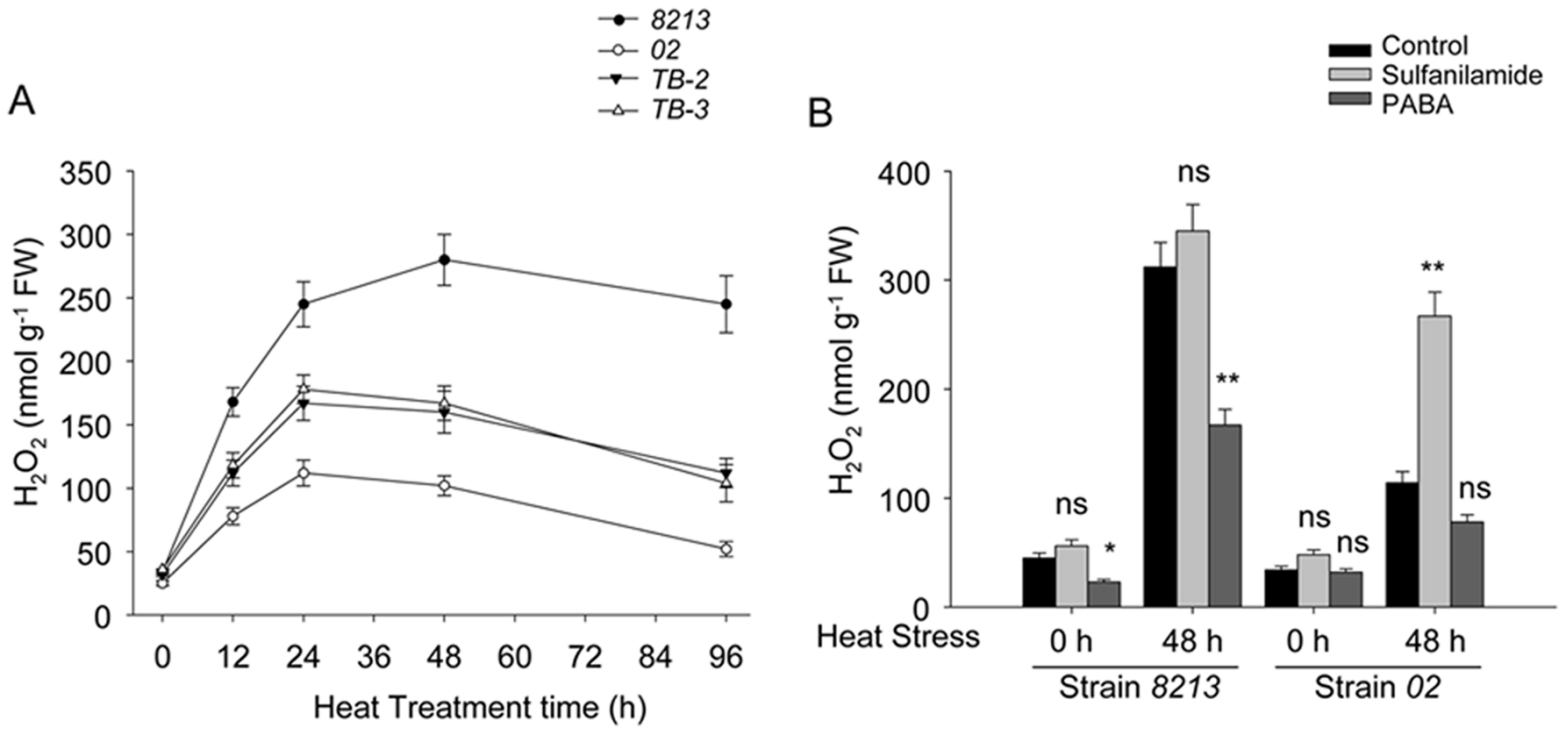
PABA reduces H_2_O_2_ accumulation in heat stressed strains *02* and *8213*. (A) Time course of accumulation of H_2_O_2_ in the mycelia of strains *02*, *8213*, *TB-2* and *TB-3* under heat stress (33°C). Strains *02*, *TB-2* and *TB-3*, which produce more PABA content than strain *8213*, have less H_2_O_2_ accumulation under parallel conditions. (B) Effects of PABA and PABA synthase inhibitor sulfanilamide on H_2_O_2_ accumulation. Strain *02* and strain *8213* were cultured on PDA medium with 1 mM PABA or 0.1 mM sulfanilamide for 6 days, then were subjected to high temperature treatment (33°C) for 48 hours as indicated, followed by H_2_O_2_ content measurement. Three independent biological replicates were performed for each analysis. Data are expressed as average ± SEM. Unpaired t-tests were performed between control sample and sulfanilamide treated sample or PABA treated sample within each strain, ns: P>0.05, *: P<0.05, **: P<0.01.

Oxidative stress usually accompanies heat stress, and could directly induce injury following heat stress in Arabidopsis [26] and yeast [27]. On the other hand, PABA could scavenge reactive oxygen species and protect DNA against free radical damage *in vitro* [28]. It is unknown whether PABA can also alleviate heat stress or oxidative stress induced damage in plants or higher fungus (e.g., mushroom). Indeed, heat stress induced fast accumulation of H_2_O_2_ in thermo-sensitive strain *8213*, while such effect was much more moderate in thermotolerant strain *02* (Figure 8A). *Pabs* over-expressing transgenic strains *TB-2* and *TB-3* showed significantly lower H_2_O_2_ as compared to their parent strain *8213* under heat stress. To directly investigate whether H_2_O_2_ could affect mushroom viability under heat stress, we treated the mushroom with 5 µM H_2_O_2_ and found that it inhibited mycelia elongation of both *02* and *8213* under heat stress. On the other hand, treatment with BHT (Butylated hydroxytoluene), an artificial scavenger of H_2_O_2_, alleviated the inhibition of mycelia elongation by heat stress (Figure S4). We further found that treatment with exogenous PABA assuaged the H_2_O_2_ accumulation in *8213* under heat stress, while treatment with the PABA synthase inhibitor sulfanilamide aggravated it in both *02* and *8213* under heat stress (Figure 8B). These data reveal a negative correlation between PABA abundance and the H_2_O_2_ accumulation in mushroom.

**Figure 9:**
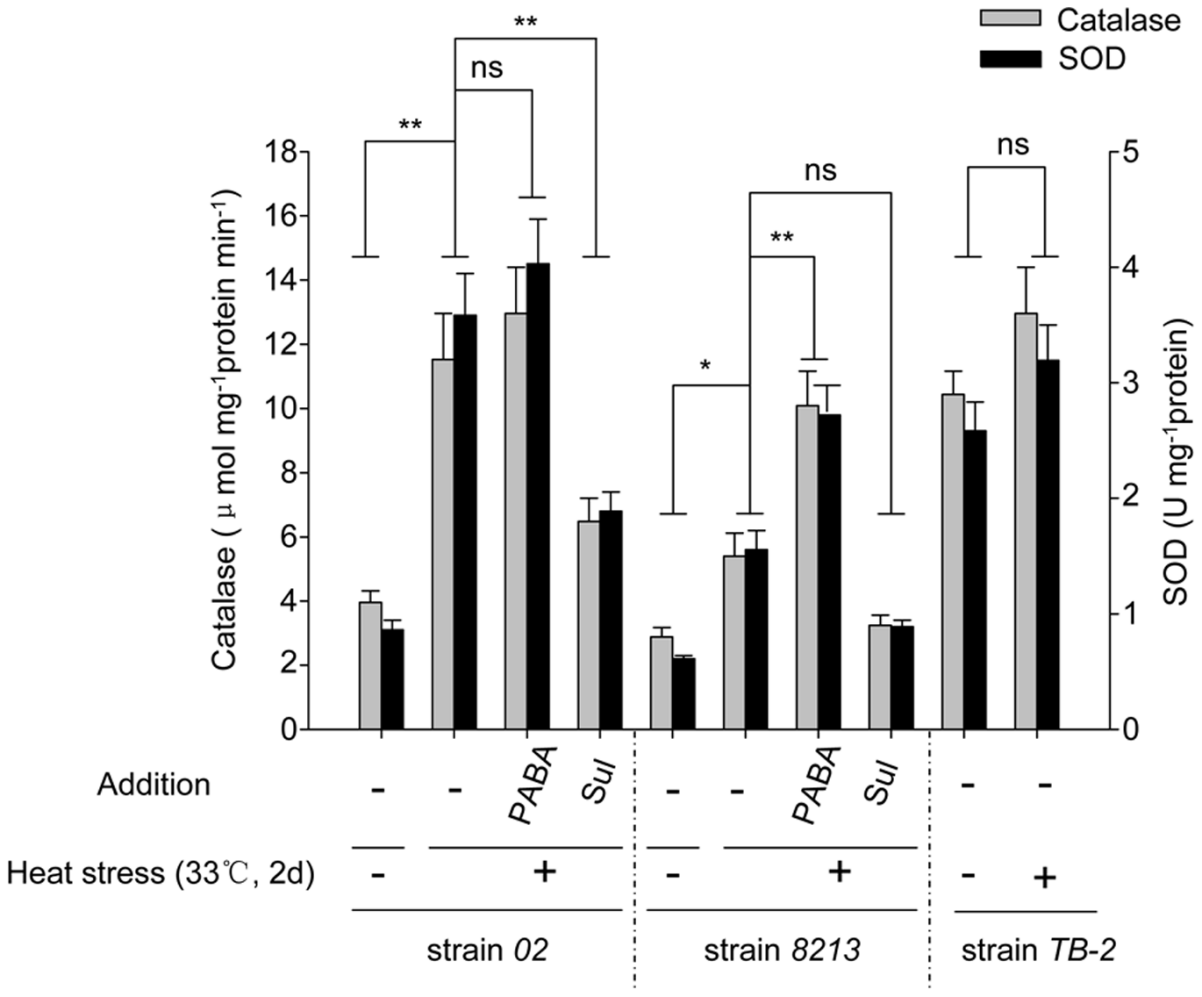
PABA increases, while sulfanilamide decreases, the activity of Catalase and SOD in *02*, *8213* and *TB-2*. Mushroom strains, as indicated, were treated with 1(33°C) for 2 days. The enzymes activities of Catalase and SOD were measured immediately after heat stress. Three independent biological replicates were performed for each analysis. Data are expressed as average ± SEM. Unpaired t-tests were performed as indicated in the figure, ns: P>0.05, *: P<0.05, **: P<0.01, Sul: sulfanilamide.

Furthermore, we found that Catalase (CAT) and Superoxide dismutase (SOD), two key antioxidants responsible for clearing oxidative free radical *in vivo*, both exhibited higher enzymatic activity in strain *02* as compared to *8213* after 2 days of heat stress (Figure 9). As a control set, addition of exogenous PABA further enhanced their enzyme activities, while treatment of sulfanilamide compromised them. As expected, the transgenic strain *TB-2*, which has higher level of basal PABA, also possesses high activity of CAT and SOD. Together, these data suggest that PABA may reduce the H_2_O_2_ accumulation through increasing CAT and SOD activity in mushroom under heat stress, thus eventually helps to alleviate the direct oxidative damaged incurred by heat stress.

### PABA Elevates HSPs and Chitinase Proteins to Enhance Thermotolerance of Mushroom under Heat Stress

Heat shock proteins (HSPs) play essential roles in organisms against high-temperature stress; and Chitinase was upregulated and contributed to the resistance against the biotic and abiotic stress in tobacco [29]. Here, we hypothesized that under heat stress, thermotolerant strain *02* should express higher level of defense-related proteins (e.g., HSPs and Chitinase), and that the induction of these proteins is mediated by PABA.

**Figure 10:**
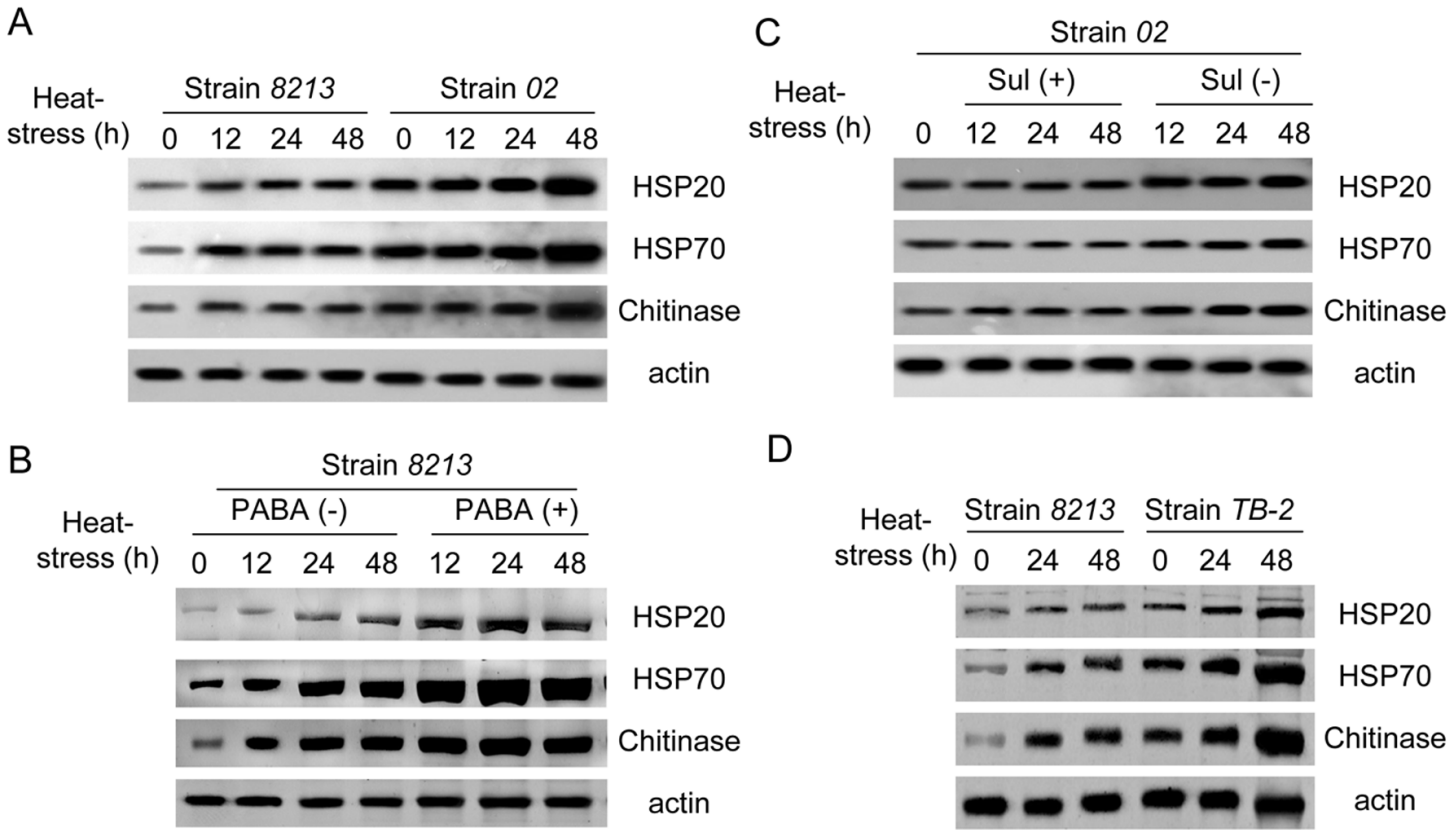
PABA mediates the accumulation of defense-related proteins in *02*, *8213* and *TB-2* under heat stress. (A) Defense-related proteins HSPs and Chitinase accumulated more in thermotolerant strain *02* than in thermo-sensitive strain *8213* under heat stress (33°C). (B) Exogenous PABA (1 mM) application increases accumulation of HSPs and Chitinase in *8213* under heat stress (33°C). (C) PABA synthase inhibitor Sulfanilamide (0.1 mM) decreases accumulation of HSPs and Chitinase in *02* under heat stress (33°C). D) *Pabs*-overexpressing transgenic line *TB-2* accumulates more HSPs and Chitinase than the parent strain *8213* under heat stress (33°C). Three independent biological replicates were performed for each sample.

We first measured the time course of expression of HSPs in strains *02* and *8213* under heat stress. As shown in Figure 10A, HSP70, HSP90 and Chitinase protein showed faster and more robust accumulation in strain *02* than in strain *8213*, with abundance peaked at 48-hour time point post-treatment. Further, we investigated whether the upregulation of those stress-resistant proteins was mediated by PABA induction or just parallel event under stress. To address this, we treated the mushrooms with either exogenous PABA or PABA synthase inhibitor sulfanilamide under heat stress to determine whether expressions of HSPs and Chitinase would change accordingly. Our results showed that application of exogenous PABA increased HSP20, HSP70 and Chitinase protein levels in thermo-sensitive strain *8213* under heat stress (Figure 10B), and inhibiting PABA production by sulfanilamide reduced the accumulations of HSPs and Chitinase in thermotolerant strain *02* (Figure 10C). Moreover, the transgenic line *TB-2* also showed higher protein accumulation of HSPs and Chitinase than strain *8213* under heat stress (Figure 10D). Together, those results demonstrate that PABA synthase and its product PABA can also induce the upregulation of HSPs and Chitinase protein upon heat stress. Thus, PABA may enhance thermotolerance of mushroom with multiple mechanisms.

## Discussion

### Comparative Proteomics Studies Unveils a Protein Network Supporting Thermotolerance of Strain *02* under Heat Stress

Mushroom *A. bisporus* strain *02* and strain *8213* have a very close phylogenetic relationship (Figure S1), and share various biological and agricultural characteristics. However, their capability to tolerate high-temperature stress is dramatically different from each other: strain *02* is thermotolerant to 33°C, which is lethal to strain *8213* (Figure 1).

To investigate the global protein changes of these two strains under heat stress, we performed a comparative proteomic analysis to identify differentially expressed proteins. First, we observed more induction of the HSPs and Chitinase protein families in strain *02* compared to strain *8213*, which may contribute to thermotolerance of stain *02*. Antioxidant enzyme proteins were also increased in strain *02* under high-temperature stress suggesting their function in clearing ROS (reactive oxygen species) in mushrooms. Accordingly, strain *02* showed lower level of H_2_O_2_ accumulation compared to *8213* under heat stress. Similarly, increasing antioxidant enzyme activities such as SOD and ascorbate peroxidase (APX) can enhance thermotolerance in potato and wheat [30], [31], [32], [33]. Meanwhile, we found that many proteins belonging to energy and metabolism groups showed higher accumulation in strain *02* than in *8213* under high temperature, which is consistent with previous studies that the proteins in the class of energy and metabolism greatly contribute to plant tolerance against high-temperature stress [34], [35], [36], [37]. The proteins from the group of cell structures and division were also induced to higher accumulation in strain *02*, which are likely responsible for the quick increase of biomass in strain *02* under high temperature. Some proteins involved in RNA folding, such as ATP-dependent RNA helicase DRS1 and ATP-dependent RNA helicase DBP8 were also differentially regulated, which indicates that they possibly modulate the RNA structure to adapt to high-temperature stress, which confirmed previous reports that RNA helicase takes part in plants’ response to abiotic stress such as cold and salt stress [38], [39]. Further, we found that Chitinase was highly induced under high-temperature stress, suggesting a function of this enzyme in mushroom thermotolerance.

Differential regulation of many proteins involved in calcium and G-protein signaling were observed in mushrooms responding to high temperature, suggesting their involvement in thermoregulation. The mitogen-activated protein kinase (MAPK) pathway is involved in plants’ response to multiple stresses [40], [41]. Consistently, we identified several protein spots that encode MEKK and related serine/threonine protein kinases (e.g., spot 17) with up-regulation in strain *02* in response to high-temperature stress.

Collectively, we propose that mushrooms use multiple strategies to enhance their tolerance against high-temperature stress.

### PABA Enhances Mushroom Thermotolerance by Removing H_2_O_2_ and Elevating Defense-related Proteins

Among the differentially expressed proteins related to heat stress, we found that a PABA synthase is constitutively more abundant in *02* and heat stress further increased its expression. We further demonstrate that mRNA level of *Pabs* gene and its synthesis product PABA correlated with the PABA synthase protein level in response to heat stress. Since PABA is structurally similar to SA, and SA is known to play a role in plants’ resistance to abiotic stress [16], [17], it’s possible that PABA may play an important role in enhancing mushroom thermotolerance. In the ensuing physiological studies, we found that exogenous PABA application could greatly improves thermotolerance of sensitive strain *8213*, as measured by reduced mycelia breaks. On the other hand, treatment with a PABA synthase inhibitor reduced the PABA accumulation and reduced thermotolerance of strain *02*. To carry our studies at a potentially applicable transgenic level, we cloned the *Pabs* gene of strain *02,* confirmed its PABA synthase activity *in vitro*, and transgenically overexpressed it in thermo-sensitive strain *8213.* These studies established that *Pabs* gene of strain *02* can substantially improve thermotolerance of strain *8213*. In addition, we found that reducing PABA synthase transcription using a transgenic antisense sequence against *Pabs* gene in strain *02* impaired its thermotolerance (data not shown). We therefore conclude that PABA modulates the mushroom’s thermotolerance.

The higher accumulation of H_2_O_2_ in strain *8213* compared to strain *02* under heat stress may explain the more severe damage in strain *8213*. We found that PABA plays a role in clearing heat stress induced H_2_O_2_ accumulation (Figure 8) likely through increasing antioxidant enzyme SOD and CAT activities (Figure 9), thus alleviating the injury. Moreover, we showed that the abundance of thermotolerance-related proteins, such as HSPs and Chitinase, was higher in strain *02* as compared to strain *8213* under heat stress. Importantly, the induction of HSPs and Chitinase can be further strengthened either through *in vitro* application of PABA or through transgenic overexpression of *Pabs* gene. PABA synthase inhibitor sulfanilamide, on the other hand, had the opposite effects. We therefore propose that PABA functions as a mediator to stimulate signaling leading to systematic acquired resistance (SAR). Future experiments using “plus and minus PABA” in proteomic profiling combined with RNA-Seq will likely identify more PABA targets and downstream pathways.

**Figure 11:**
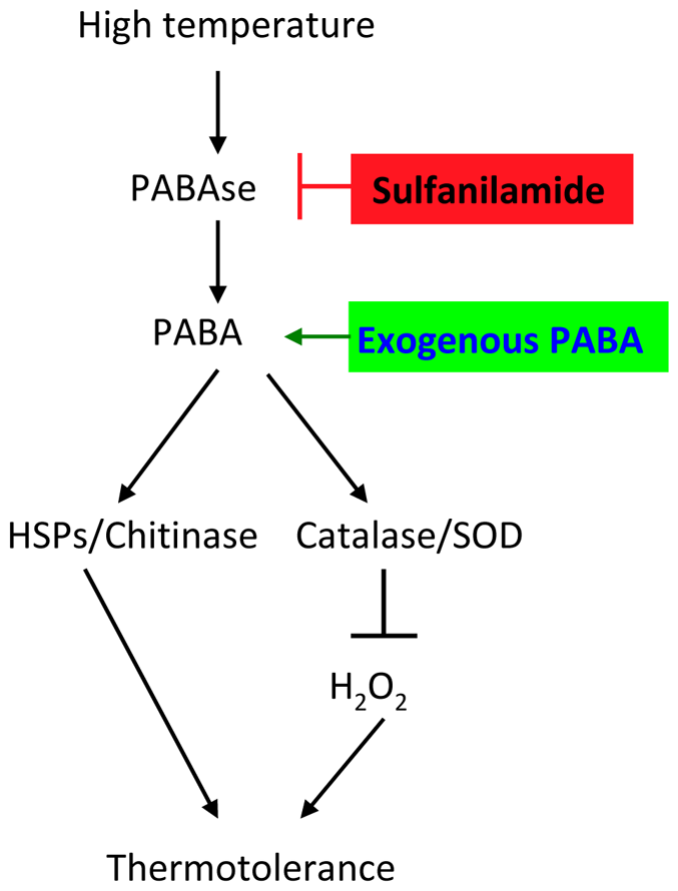
Schematic model for the role of PABA in enhancing thermotolerance of mushroom. See Discussion for details.

To summarize, we propose a model for the role of PABA in enhancing mushroom thermotolerance through mediating the expression of defense-related effecters and clearing H_2_O_2_ accumulation (Figure 11). Mushrooms have adopted multiple strategies to adapt to heat stress; important among these is the upregulation of PABA. Experimentally increasing PABA level enhances, while reducing PABA level impairs, mushroom’s tolerance against heat stress. Our findings provide new insight into the role of PABA in enhancing thermotolerance of mushroom.

## Supporting Information

Figure S1 **Phylogenetic trees of strains** ***02*** **and** ***8213*** **as well as closely related species in the genus** ***Agaricus*** **constructed with ITS1, ITS2, and 5.8S subunit DNA sequences.**
(TIF)

Figure S2 **Proteomic profiling of mushroom strain** ***02*** **and strain** ***8213*** **with or without high-temperature stress (33°C/24 h).**
(TIF)

Figure S3 **Purification of PABA synthase protein (encoded by Pabs) from** ***E. coli*** **.**
(TIF)

Figure S4 **Effects of H_2_O_2_ and BHT (Butylated hydroxytoluene) on mycelia elongation in strains** ***02*** **and** ***8213*** **under heat stress.**
(TIF)

Table S1 **Identification of high temperature induced proteins in** ***Agaricus bisporus*** **by MS/MS analysis.**
(DOC)
