# Supplementary figures and images for: Para-Aminobenzoic Acid (PABA) Synthase Enhances Thermotolerance of Mushroom Agaricus bisporus
Source: PLoS One. 2014 Mar 10;9(3):e91298. doi: 10.1371/journal.pone.0091298 (PMC3948851; doi:10.1371/journal.pone.0091298)

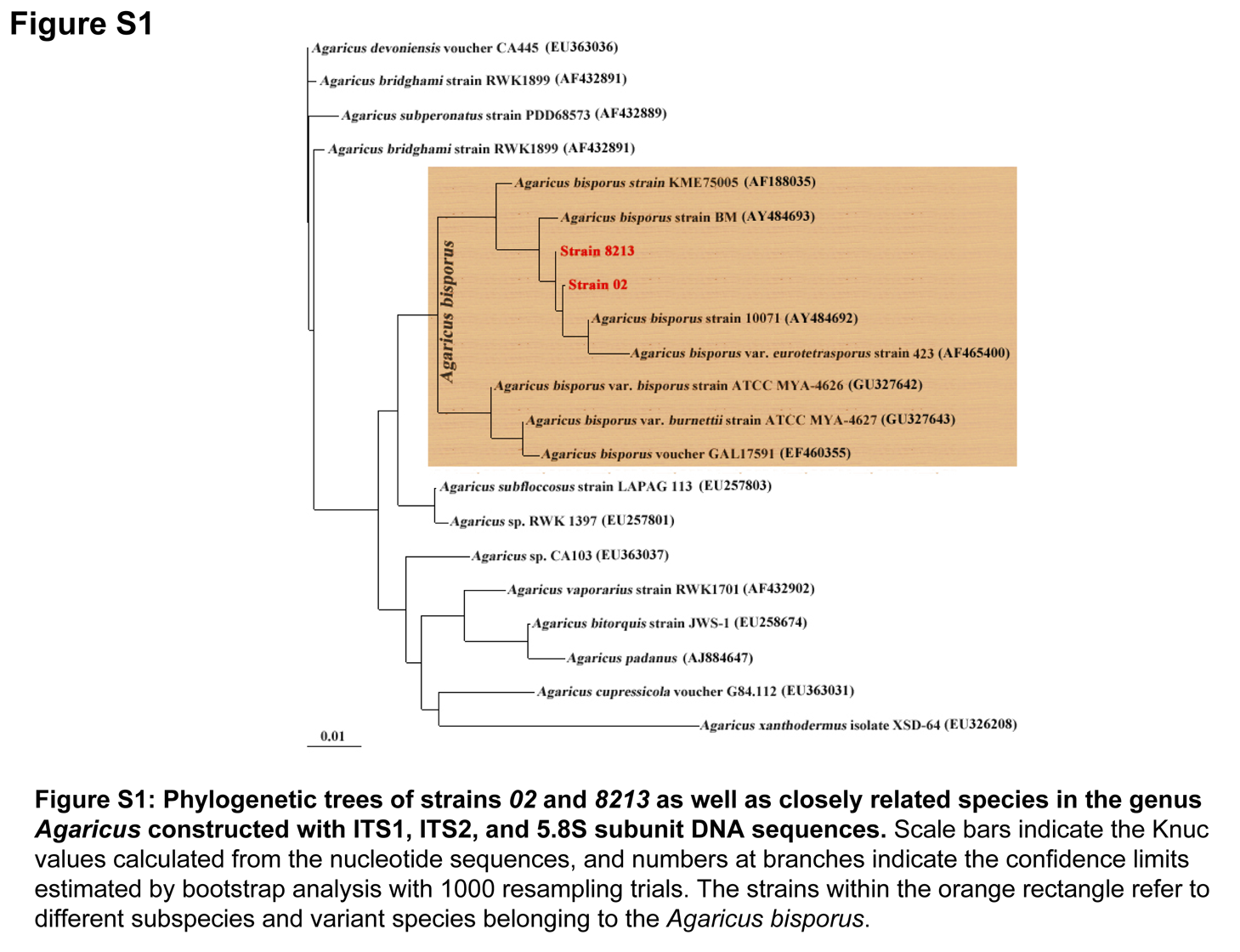

Supplement: Figure S1 — Phylogenetic trees of strains 02 and 8213 as well as closely related species in the genus Agaricus constructed with ITS1, ITS2, and 5.8S subunit DNA sequences. (TIF) [file pone.0091298.s001.tif]

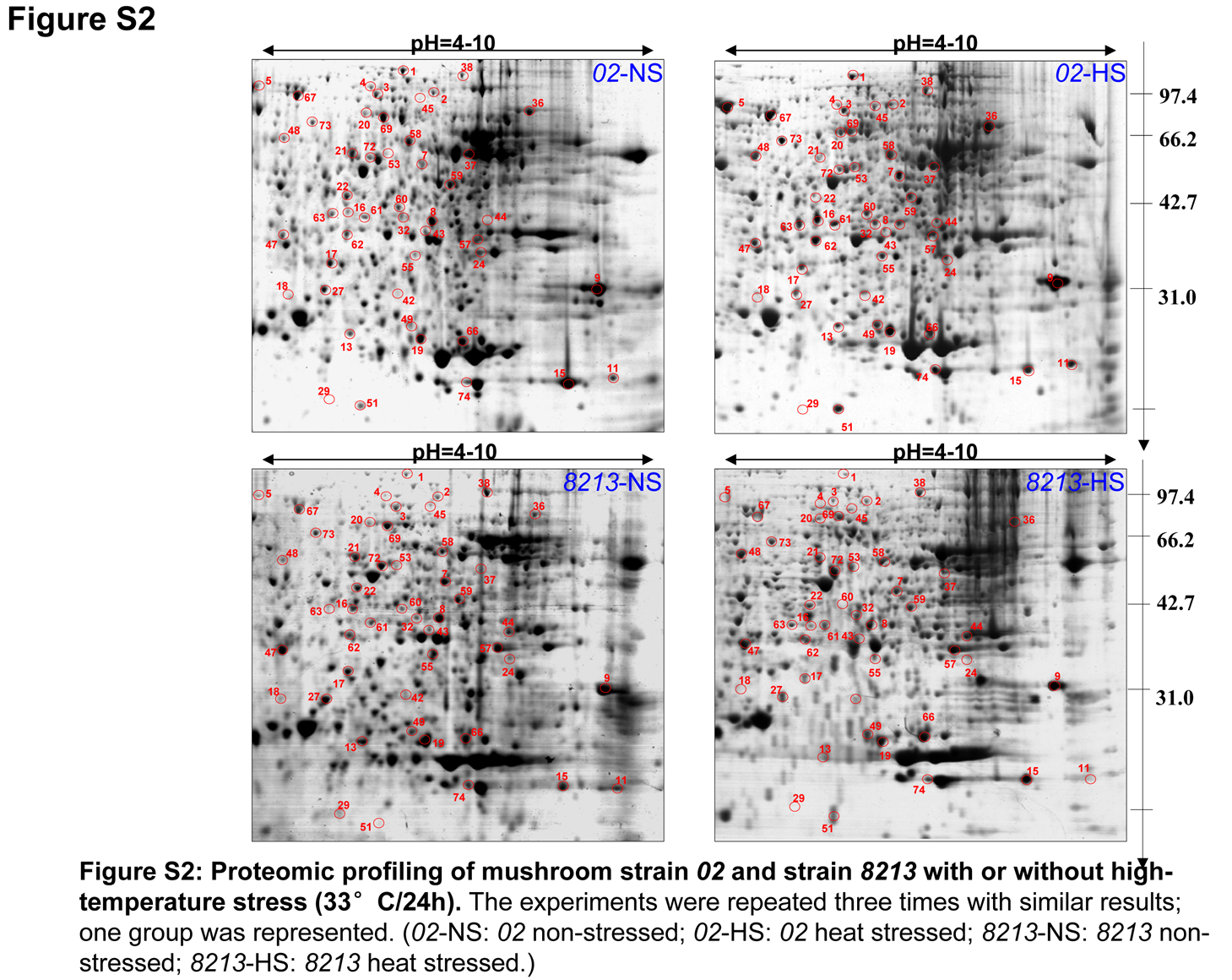

Supplement: Figure S2 — Proteomic profiling of mushroom strain 02 and strain 8213 with or without high-temperature stress (33°C/24 h). (TIF) [file pone.0091298.s002.tif]

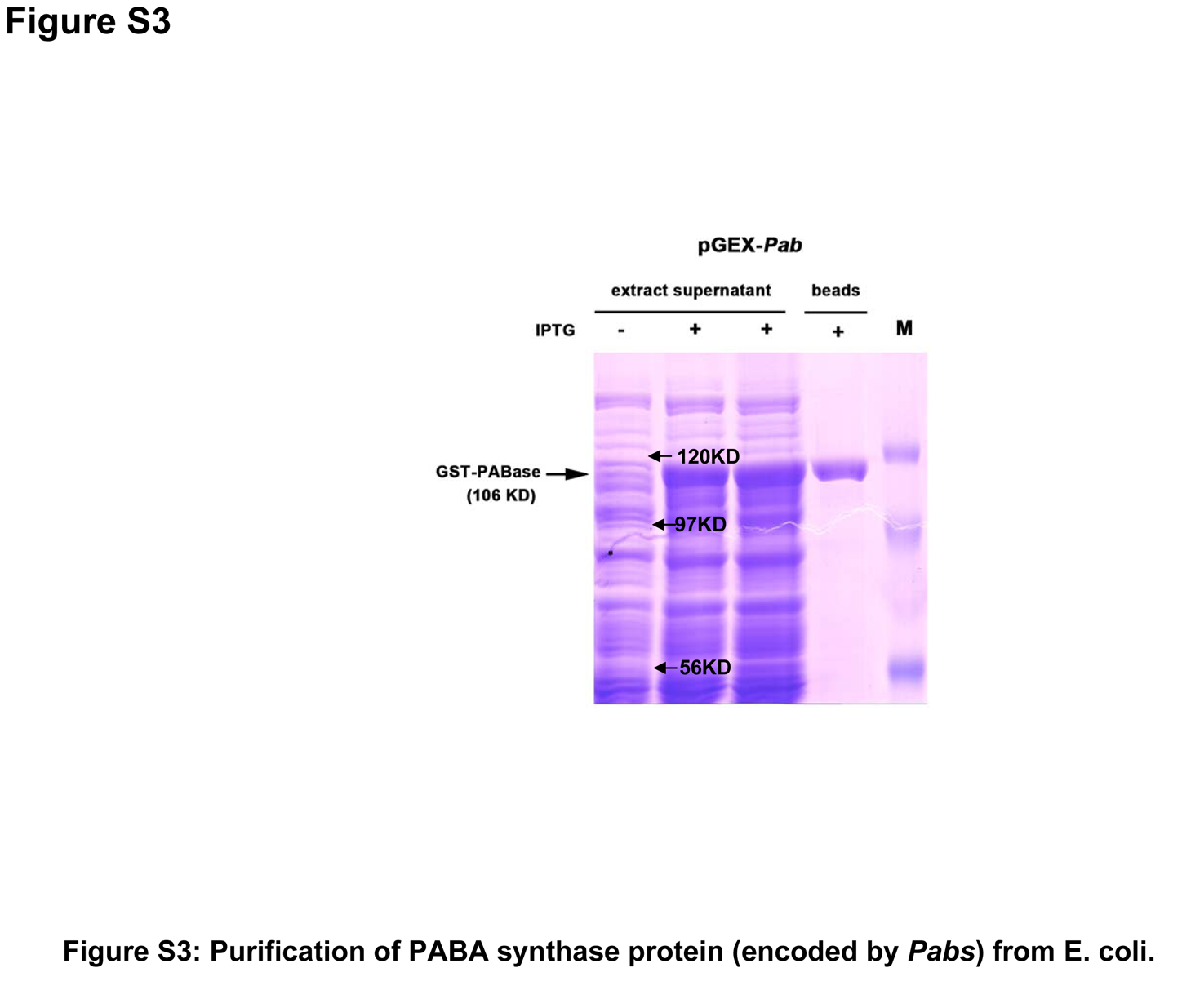

Supplement: Figure S3 — Purification of PABA synthase protein (encoded by Pabs) from E. coli . (TIF) [file pone.0091298.s003.tif]

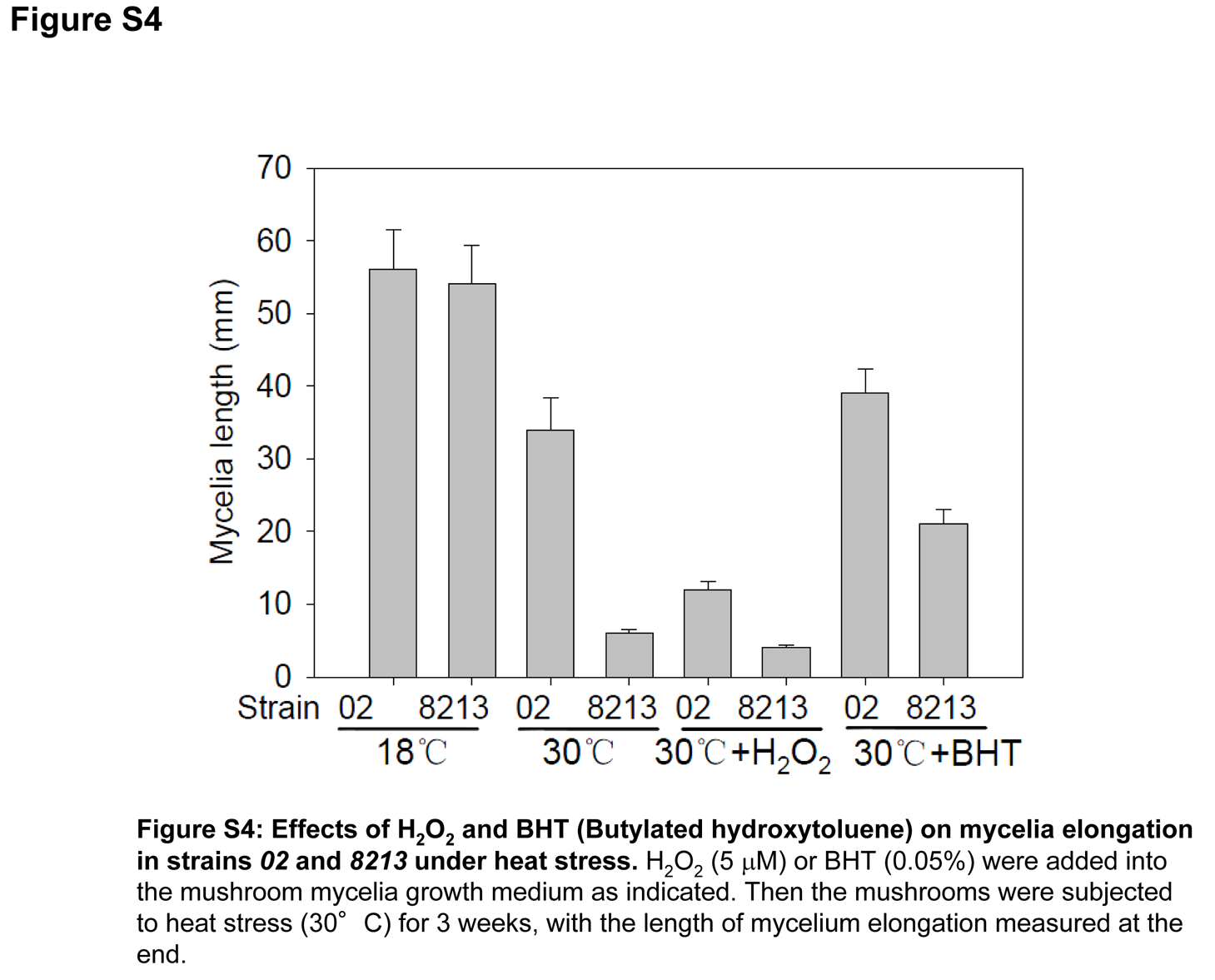

Supplement: Figure S4 — Effects of H2O2 and BHT (Butylated hydroxytoluene) on mycelia elongation in strains 02 and 8213 under heat stress. (TIF) [file pone.0091298.s004.tif]
