# Supplementary material for: Para-Aminobenzoic Acid (PABA) Synthase Enhances Thermotolerance of Mushroom Agaricus bisporus
Source: PLoS One. 2014 Mar 10;9(3):e91298. doi: 10.1371/journal.pone.0091298 (PMC3948851; doi:10.1371/journal.pone.0091298)
Supplement: Table S1 — Identification of high temperature induced proteins in Agaricus bisporus by MS/MS analysis. (DOC) [file pone.0091298.s005.doc]

Table S1: Identification of high temperature induced proteins in *Agaricus bisporus* by MS/MS analysis.

| Spots NO. | NCBI Accession NO.^a^ | Protein name | Exp. Mw/Pi^b^ | Theo. Mw/pI^c^ | Score^d^ | C(%)^e^ | Ratio^f^ | | | Sequence^g^ |
| --- | --- | --- | --- | --- | --- | --- | --- | --- | --- | --- |
|  |  |  |  |  |  |  | 02-HS  /02-NS | 8213-NS/02-NS | 8213-HS  /02-NS |  |
| Energy and metabolism pathway | | | | | | | | | | |
| 3 | [183828](http://genome.jgi-psf.org/cgi-bin/dispGeneModel?db=Agabi_varbisH97_2&id=183828) | Urease | *90.3/6.1* | 90.6/5.7 | 134 | 8.6% | 3.73 | 0.76 | 0.88 | ALSFDRLK  NCLDVADK  WNDTTPSMK  EGMGQATNR  SIPEDLDFAESR  VLLHQVGFIAQK  TEAVALIASVLQER |
| 15 | [195361](http://genome.jgi-psf.org/cgi-bin/dispGeneModel?db=Agabi_varbisH97_2&id=195361) | SNARE protein YKT6 | *23.2/8.2* | 22.3/8.2 | 156 | 22.1% | 2.14 | 2.56 | 6.69 | TAQTQR  LLDDFTAK  VQQELDETK  GSVGEFLSFFTK |
| 19 | [191204](http://genome.jgi-psf.org/cgi-bin/dispGeneModel?db=Agabi_varbisH97_2&id=191204) | Exosomal 3'-5' exoribonuclease complex | *28.3/6.7* | 28.4/6.6 | 98 | 17.3% | 2.15 | 3.79 | 2.95 | EAKMR  LAGEAGK  VALVTMETR  VEILNDGGYR  STFEPVVQTTLYPR |
| 24 | [198583](http://genome.jgi-psf.org/cgi-bin/dispGeneModel?db=Agabi_varbisH97_2&id=198583) | Glyoxylate/hydroxypyruvate reductase | *35.5/7.3* | 35.62/7.3 | 144 | 17.8% | 1.77 | 0.45 | 0.67 | AGNVQWQK  GIDVTHAK  PTAYLVNTSR  VIAWSPHLTK  AFWGQTVENIK  LYDYEIICAMR |
| 27 | [192128](http://genome.jgi-psf.org/cgi-bin/dispGeneModel?db=Agabi_varbisH97_2&id=192128) | Fructose-bisphosphate aldolase | *32.5/5.5* | 39.2/5.9 | 151 | 10.6% | 0.07 | 1.74 | 0.04 | EENIK  YYDPR  GLANDK  PELLAK  TLSERVK  LFDYAKANK |
| 29 | [205090](http://genome.jgi-psf.org/cgi-bin/dispGeneModel?db=Agabi_varbisH97_2&id=205090) | Flavodoxin/nitric oxide synthase | *21.3/5.6* | 21.6/5.6 | 204 | 16.7% | 0.13 | 0.03 | 0.08 | MSPPK  LEVTIAEK  HGIMPAPWK  AFAEMTSFEMIR |
| 36 | 192907 | para-aminobenzoic acid synthetase | *80.7/5.6* | 81.2/5.6 | 135 | 7.3% | 3.17 | 0.29 | 0.49 | YHSLHIR  DVWTFFDEYR  EIAEEMLAGSR  LASDWNTINR  DTPEDVDDSLGWEVLR |
| 42 | [187748](http://genome.jgi-psf.org/cgi-bin/dispGeneModel?db=Agabi_varbisH97_2&id=187748) | Inosine guanosine and xanthosine phosphorylase family protein | *32.6/6.3* | 32.4/6.1 | 91 | 11.8% | 0.99 | 1.11 | 0.14 | EEGLK  AEVIKSLVQR  SELAFGLLGK  TNILSTVEYIR |
| 43 | [192003](http://genome.jgi-psf.org/cgi-bin/dispGeneModel?db=Agabi_varbisH97_2&id=192003) | Sorbitol dehydrogenase | *39.5/6.6* | 58.3/6.9 | 127 | 11.1% | 0.88 | 0.39 | 0.31 | YEQR  ALAAK  LEFAK  PMEGESK  VAMEPGATCK  VDLKPLVTHR |
| 47 | [201886](http://genome.jgi-psf.org/cgi-bin/dispGeneModel?db=Agabi_varbisH97_2&id=201886) | Phosphatidylinositol transfer protein PDR16 and related proteins | *37.5/4.9* | 37.8/4.8 | 221 | 15.7% | 1.54 | 1.54 | 0.41 | WMGKWR  SEMAIER  NTSLSVAR  PTFYMIPSR  FWLSYECILR  QIHYTVWLLER |
| 48 | 120553 | Aspartyl beta-hydroxylase | *60.6/4.9* | 60.2/5.2 | 167 | 11.2% | 1.13 | 0.44 | 0.48 | INLGSR  TPYPSR  CLIAWLYR  LSTSQNPYR  WLCQHDDK  SNTPYVFSAR  SLDSTPLDGPTR |
| 49 | [120270](http://genome.jgi-psf.org/cgi-bin/dispGeneModel?db=Agabi_varbisH97_2&id=120270) | Phosphoglycerate mutase | *28.8/6.5* | 28.4/6.1 | 190 | 15.9% | 0.72 | 1.25 | 0.29 | SIEELSK  TIWAGWK  PPLVVTPK  LDPEVEQLK  AHSTALAVLR |
| G-protein signal | | | | | | | | | | |
| 44 | [211352](http://genome.jgi-psf.org/cgi-bin/dispGeneModel?db=Agabi_varbisH97_2&id=211352) | G-protein alpha subunit (small G protein superfamily) | *40.4/7.2* | 40.5/7.2 | 78 | 16.3% | 1.67 | 2.73 | 3.13 | PNSK  EAISR  LNEDESVNR  EIILANTIQSLR  LELQVSDANITHR  IYAHYICATDPQQAK |
| 72 | [121576](http://genome.jgi-psf.org/cgi-bin/dispGeneModel?db=Agabi_varbisH97_2&id=121576) | Rac GTPase-activating protein BCR/ABR | *58.5/6.2* | 58.1/6.26 | 89 | 10.1% | 1.13 | 2.65 | 4.65 | QDVEK  GLEDMLR  EAETPHDQR  TQSPRAHSR  MLTSSSLHER  SDNYEYDSEQYNR |
| Antioxidant enzyme system | | | | | | | | | | |
| 16 | [135514](http://genome.jgi-psf.org/cgi-bin/dispGeneModel?db=Agabi_varbisH97_2&id=135514) | Glutamine synthetase | *40.6/5.8* | 39.5/5.9 | 115 | 11.0% | 3.72 | 1.87 | 0.67 | GASIR  YLLVR  VFDEAK  DLIEAHYR  IQAEYVWIDGDGGLR |
| 32 | [175594](http://genome.jgi-psf.org/cgi-bin/dispGeneModel?db=Agabi_varbisH97_2&id=175594) | Iron/ascorbate family oxidoreductases | *39.3/6.5* | 39.1/6.4 | 158 | 14.2% | 0.21 | 0.11 | 0.11 | SNMHR  DPQEIDR  PGNSKVLR  DTVEFWNISK  NHEAEEEVAK  NPPMPHNDQK |
| 37 | [186880](http://genome.jgi-psf.org/cgi-bin/dispGeneModel?db=Agabi_varbisH97_2&id=186880) | Cytochrome P450 CYP2 subfamily | *58.6/7.2* | 59.2/7.3 | 189 | 11.9% | 1.78 | 0.35 | 0.47 | AWLEYDK  PGLDNNGK  YTFADGILK  IIPEWFPGAEWK  VFGQGFLVLGSVK  ENPNNLPPPPGPK |
| 55 | [122853](http://genome.jgi-psf.org/cgi-bin/dispGeneModel?db=Agabi_varbisH97_2&id=122853) | Defense-related protein containing SCP domain | *36.3/6.6* | 37.2/6.6 | 104 | 8.1% | 1.22 | 0.78 | 0.32 | MALFVR  PGSYQPK  VSSPTSLR  SPAARNSVR |
| 59 | [139240](http://genome.jgi-psf.org/cgi-bin/dispGeneModel?db=Agabi_varbisH97_2&id=139240) | cystathionine gamma-synthases | *46.6/6.9* | 46.4/6.8 | 112 | 13.1% | 1.00 | 0.34 | 0.29 | FDWVK  VVDGLK  HFGIGVK  MANPFYK  QNGFIYSR  AIFIESIANPK  EQLESGVTPDLIR |
| 66 | [204991](http://genome.jgi-psf.org/cgi-bin/dispGeneModel?db=Agabi_varbisH97_2&id=204991) | Glutathione S-transferase | *28.8/7.1* | 29.16/7.1 | 107 | 19.1% | 2.25 | 0.82 | 0.55 | EVNPYK  LVAQVK  IWIALEER  EHYGQIYGR  SQAGEAWTK  PSLLPAEPYDR |
| 67 | [115586](http://genome.jgi-psf.org/cgi-bin/dispGeneModel?db=Phchr1&id=122440) | Catalase | *92.4/5.2* | 83.2/5.5 | 204 | 7.1% | 1.38 | 0.35 | 0.27 | AIHWIR  DILDATK  VTYDAK  ELDGLTER  NIDLDLAK  IPDTDNWLK  FPDFVHALK |
| 74 | 194955 | Manganese and iron superoxide dismutase | *23.2/6.8* | 24.6/7.2 | 211 | 13.5% | 9.92 | 1.32 | 7.49 | TALR  GLAAR  LLAAAK  LGDGPLK  INLQSALK |
| Defense response protein | | | | | | | | | | |
| 7 | [193217](http://genome.jgi-psf.org/cgi-bin/dispGeneModel?db=Agabi_varbisH97_2&id=193217) | Molecular chaperone (DnaJ superfamily) | *55.7/6.8* | 55.1/6.7 | 96 | 11.4% | 5.56 | 0.12 | 0.08 | AAMK  AWLTK  EGTEIK  GLIEMGR  VSTSEAR  NPSPEAEEK  GDVDEEGLR  DPAQAHAQR |
| 9 | [201136](http://genome.jgi-psf.org/cgi-bin/dispGeneModel?db=Agabi_varbisH97_2&id=201136) | chitinase | *45.5/8.4* | 45.5/8.5 | 99 | 8.9% | 1.79 | 2.91 | 4.68 | VSSR  TCQSR  ASGASK  LSSIAR  VTFNGHLWQNK |
| 38 | [118459](http://genome.jgi-psf.org/cgi-bin/dispGeneModel?db=Agabi_varbisH97_2&id=118459) | Molecular chaperone (DnaJ superfamily) | *105.5/7.2* | 105.4/7.1 | 112 | 12.1% | 0.93 | 1.52 | 0.19 | TAQR  EDPSNK  FDYEHR  SGPFALEK  ASQYTDYTK  EDLVIDVECR  SAQVVCNFLR  SDAQEIGHTR  QGDHDYFNNAR  QQQQHNMVWVR  EPSELQISQYQK  YNSNLPFLWLICSR |
| 51 | [188751](http://genome.jgi-psf.org/cgi-bin/dispGeneModel?db=Agabi_varbisH97_2&id=122146) | Heat shock protein 20 | *20.0/6.1* | 16.24/6.1 | 85 | 21.1% | 1.32 | 0.40 | 0.48 | LPQGIK  PFGISR  LSPDQEPK  LDLHEDTQK |
| 60 | [65345](http://genome.jgi-psf.org/cgi-bin/dispGeneModel?db=Agabi_varbisH97_2&id=65345) | Molecular chaperone (DnaJ superfamily) | *42.6/6.4* | 42.6/6.45 | 154 | 12.9% | 0.48 | 0.50 | 0.17 | DAGNWR  ILDVDK  DPDAESK  NLTHLDK  VLCDHCR  YGEDALK  GEGMPDLEK |
| 61 | [194815](http://genome.jgi-psf.org/cgi-bin/dispGeneModel?db=Agabi_varbisH97_2&id=194815) | HSP90 co-chaperone CPR7/Cyclophilin | *40.2/6.1* | 40.6/6.0 | 150 | 18.0% | 1.65 | 0.54 | 0.56 | VIK  ELGNK  GMAHGILK  FEDEAFPVQHTK  PITYFDISIGDK  TAENFRALCTGEK  SIRYLDTHQEVPEK |
| 62 | [216197](http://genome.jgi-psf.org/cgi-bin/dispGeneModel?db=Agabi_varbisH97_2&id=216197) | Chaperonin GroEL (HSP60 family) | *37.8/5.8* | 33.3/5.6 | 119 | 11.2% | 3.80 | 0.79 | 0.67 | NMGR  MLQK  AISGAVR  LNILVVK  IDDYVQAR  AASTSLNSK  LLSQYAHTLK  APTQAQQGVTK |
| 73 | 192819 | Heat shock protein 70 | *70.2/5.6* | 72.2/5.8 | 137 | 6.5% | 3.81 | 0.81 | 1.33 | LIGR  IFQGER  VQDTVK  DQLDASEK  STGEEYANK  YAVIPPSMTR |
| Transcriptional factor | | | | | | | | | | |
| 2 | [185326](http://genome.jgi-psf.org/cgi-bin/dispGeneModel?db=Agabi_varbisH97_2&id=185326) | translation initiation factor | 97.4/6.6 | 90.2/6.5 | 189 | 12.0% | 1.93 | 0.22 | 0.23 | LTATK  TLDSK  EQLQER  QDEPLGR  TVTSIISK  GSIISFIDR  FQTLSPEQEK  QTTSTTTSTK  VVQFTSEGVLK  TIVQLGLCAFR  DESDEEESDDEDK |
| 5 | [182716](http://genome.jgi-psf.org/cgi-bin/dispGeneModel?db=Agabi_varbisH97_2&id=182716) | MADS box transcription factor | *93.3/4.2* | 94.3/4.2 | 173 | 8.5% | 3.26 | 1.32 | 5.25 | EAMDK  LQASLR  ALGQGDK  IWIYCSR  VPQPGPR  AYIPVDIAK  LQPGFNFPR  PIRAPILMR  SAVGEAATEESK |
| 45 | [118399](http://genome.jgi-psf.org/cgi-bin/dispGeneModel?db=Agabi_varbisH97_2&id=118399) | Fungal transcriptional regulatory protein | *96.7/6.5* | 97.3/6.4 | 135 | 8.9% | 1.46 | 0.61 | 0.31 | ITHLTTR  IPCQSCLR  SHLPPWNR  AALALQPILEK  FASECVAEVTSR  ETQQPSSSSQHTR  ILFWDLFVADVWQSLNTGR |
| Protein kinase | | | | | | | | | | |
| 8 | [200781](http://genome.jgi-psf.org/cgi-bin/dispGeneModel?db=Agabi_varbisH97_2&id=200781) | Phosphotyrosyl phosphatase activator | *40.4/6.6* | 41.2/6.6 | 169 | 16.5% | 2.77 | 0.80 | 1.37 | INTVKR  FGNLAFR  TDEDIER  MYKAEVLGK  LCWKLQDVYK  SYYDYDLFLR  VDDLLEQFQIR |
| 13 | [69119](http://genome.jgi-psf.org/cgi-bin/dispGeneModel?db=Agabi_varbisH97_2&id=69119) | Phosphomevalonate kinase | *28.2/6.1* | 28.3/6.2 | 96 | 15.2% | 4.07 | 0.11 | 0.23 | LAVELK  MIKPFNK  FVQLALEK  SGITLSDVHK  MPLSTVVSSPGK |
| 17 | [117497](http://genome.jgi-psf.org/cgi-bin/dispGeneModel?db=Agabi_varbisH97_2&id=117497) | MEKK and related serine/threonine protein kinases | *34.4/5.6* | 33.8/5.5 | 79 | 17.9% | 2.65 | 1.17 | 1.43 | VALDK  GELVVVK  AENVLIDR  GQLNQMEELR  STHNVSFCWK  VVIAINIGEIPSR |
| 18 | [115147](http://genome.jgi-psf.org/cgi-bin/dispGeneModel?db=Agabi_varbisH97_2&id=115147) | Serine/threonine protein phosphatase | *31.5/5.0* | 31.3/4.9 | 137 | 14.4% | 1.79 | 2.56 | 1.98 | MLSRPK  VSPFER  LPANSGR  TQTHFFNCPK  EDDVTVIVGLIK |
| 21 | [222826](http://genome.jgi-psf.org/cgi-bin/dispGeneModel?db=Agabi_varbisH97_2&id=222826) | Dihydroxyacetone kinase 1 | *61.4/5.82* | 63.2/5.8 | 145 | 11.5% | 0.52 | 0.11 | 0.21 | TVFVAK  SFIPFK  LADAVR  PDVPGWK  AGAEAILK  SAYVEGDK  GIDLIDNEK  GGSLDEVYK  AVDPDAFVK |
| 53 | [121526](http://genome.jgi-psf.org/cgi-bin/dispGeneModel?db=Agabi_varbisH97_2&id=121526) | Serine/threonine protein kinase | *55.5/6.2* | 54.9/6.3 | 105 | 8.9% | 1.52 | 1.43 | 0.37 | TGSR  SDHANGFR  SEVHVWLSR  FLEAANQQPR  VIVELMDMIVTHK |
| 58 | [122140](http://genome.jgi-psf.org/cgi-bin/dispGeneModel?db=Agabi_varbisH97_2&id=122140) | Histidine acid phosphatase | *62.4/6.7* | 61.6/6.6 | 114 | 11.4% | 2.08 | 0.79 | 0.95 | YEEDK  VTIQER  VSLSDR  NGDYVR  HTFPLDK  LPELADQK  SNGNGDFDR  LNSAAIDLPENLTAK |
| 69 | [228705](http://genome.jgi-psf.org/cgi-bin/dispGeneModel?db=Agabi_varbisH97_2&id=228705) | Serine/threonine protein kinase | *77.3/6.2* | 76.67/6.1 | 136 | 8.4% | 3.79. | 0.91 | 0.68 | YQDNR  QPNRR  FVCLEK  TLTAVFR  VIYAHSDILTR  QLSSPYSPLPSR  QWTFTGLEWTVR |
| Cell division and structure protein | | | | | | | | | | |
| 1 | [186557](http://genome.jgi-psf.org/cgi-bin/dispGeneModel?db=Agabi_varbisH97_2&id=186557) | RNA-binding protein | *110.5/6.22* | 110.3/6.2 | 177 | 9.4% | 2.34 | 0.21 | 0.11 | EAITR  SQANR  DWELER  PPADYEK  HLQFIIDR  AQYFHSER  TIAETGLTR  AGGMFVPRK  SGEAGFVEELR  SVPGSGEVWAR  ISEQEVGVHLAWK |
| 4 | [185326](http://genome.jgi-psf.org/cgi-bin/dispGeneModel?db=Agabi_varbisH97_2&id=185326) | Eukaryotic translation initiation factor | *97.3/6.12* | 97.9/6.1 | 99 | 7.9% | 2.39 | 0.32 | 0.22 | ALTAMK  EQLQER  ILSAQER  LLDFADR  VLLALISSR  GSIISFIDR  QTTSTTTSTK  FSQGLGNQMGQITQR |
| 11 | [182852](http://genome.jgi-psf.org/cgi-bin/dispGeneModel?db=Agabi_varbisH97_2&id=182852) | Transport protein particle (TRAPP) complex subunit | *24.4/8.9* | 24.5/8.8 | 114 | 20.6% | 2.44 | 4.34 | 8.91 | ESAR  ASAAVATAR  AVVDEDEEALR  GMFSDTLDIIK  IYAAMPAGVIK |
| 20 | [193780](http://genome.jgi-psf.org/cgi-bin/dispGeneModel?db=Agabi_varbisH97_2&id=193780) | Concanavalin A-like lectin/glucanase | *75.2/6.1* | 75.7/6.1 | 146 | 9.3% | 3.11 | 0.57 | 1.26 | TGNDGK  APPAQSGR  PTSDYIQK  DAGVYTNSR  LVITMTEKR  DPDLDDALHDPR  DGGQLSGLPGQK |
| 22 | [191675](http://genome.jgi-psf.org/cgi-bin/dispGeneModel?db=Agabi_varbisH97_2&id=191675) | Actin-related protein | *48.4/5.8* | 47.6/5.7 | 130 | 12.4% | 0.28 | 1.35 | 0.35 | SIVGQR  ACDAELR  LSNELTR  AGYAGDDTPR  LYIGQSGPSIWR  TTETWQSWAEER |
| 57 | [194378](http://genome.jgi-psf.org/cgi-bin/dispGeneModel?db=Agabi_varbisH97_2&id=194378) | Mitotic spindle checkpoint protein BUB3 | *38.2/7.1* | 37.2/7.1 | 137 | 13.8% | 1.52 | 2.48 | 2.36 | FDHR  QYPK  ELDLSTEK  NMSSPTQQR  TAQQPMIVIR  LLVSSWDTTVR |
| 63 | [207822](http://genome.jgi-psf.org/cgi-bin/dispGeneModel?db=Agabi_varbisH97_2&id=207822) | ATP synthesis coupled proton | *39.5/5.6* | 40.1/5.5 | 188 | 13.8% | 3.23 | 0.38 | 0.79 | DRYVK  AADVREDDPR  PSLEFQQEQER  SQFDQVNANLR  TLSPLVQEDYDK |

a, Database accession numbers according to NCBInr

b, Theoretical Mw/pI

c, Experimental Mw/pI

d, The Mascot search score against the database of NCBInr

e, Sequences coverage

f, protein spots showed a significant change in abundance( fold change) by a factor>2-fold compared to the control analyzed by LSD test. A p-value of <0.05 was considered statistically significant

g, the identified peptide sequences by MS/MS
